# Deciphering the functional role of hypothetical proteins from *Chloroflexus aurantiacs* J-10-f1 using bioinformatics approach

**DOI:** 10.22099/mbrc.2020.36894.1495

**Published:** 2020-09

**Authors:** Chander Jyoti Thakur, Sandeep Saini, Aayushi Notra, Bhavanshu Chauhan, Sarthak Arya, Rishabh Gupta, Jyotsna Thakur, Varinder Kumar

**Affiliations:** 1 Department of Bioinformatics, GGDSD College, Sector 32-C, 160030, Chandigarh, India; 2 Department of Biophysics, Panjab University, Sector 25, 160014, Chandigarh, India

**Keywords:** Domains, Physicochemical properties, Thermophilic proteins, Carbohydrate metabolism

## Abstract

*Chloroflexus aurantiacus *J-10-f1 is an anoxygenic, photosynthetic, facultative autotrophic gram negative bacterium found from hot spring at a temperature range of 50-60°C. It can sustain itself in dark only if oxygen is available thereby exhibiting a dark orange color, however display a dark green color when grown in sunlight. Genome of the organism contains total of 3853 proteins out of which 785 (~20%) proteins are uncharacterised or hypothetical proteins (HPs). Therefore in this work we have characterized the 785 hypothetical proteins of *Chloroflexus aurantiacus *J-10-f1 using bioinformatics tools and databases. HPs annotated by more than five domain prediction tools were filtered and named high confidence-hypothetical proteins (HC-HPs). These HC-HPs were further annotated by calculating their physiochemical properties, homologous, subcellular locations, signal peptides and transmembrane regions. We found most of the HC-HPs were involved in photosynthesis, carbohydrate metabolism, biofuel production and cellulose synthesis processes. Furthermore, few of these HC-HPs could provide resistance to bacteria at high temperature due to their thermophilic nature. Hence these HC-HPs have the potential to be used in industrial as well as in biomedical needs. To conclude, the bioinformatics approach used in this study provides an insight to better understand the nature and role of *Chloroflexus aurantiacus *J-10-f1 hypothetical proteins.

## INTRODUCTION

The thermophilic bacterium *Chloroflexus aurantiacus *(gram -ve) was the first filamentous anoxygenic phototropic (FAP) bacterium which occupy an intermediate position between 'classical' green bacteria (*Chlorobiaceae*) and the purple photosynthetic bacteria [1, 2]. Being earliest photosynthetic facultative autotrophic bacteria, Chloroflexi species are considered popular model organism for studying anoxygenic photosynthesis and autotrophic CO2 assimilation as well as attract the attention of researchers to explore heat stable enzymes which are useful for bioengineering and biotechnology [3, 4].

Anoxygenic photosynthesis helps to understand the evolution of photosynthesis that contain B808-866 light-harvesting core complex, quinone-type (or type-II) reaction center and  peripheral antenna complex known as a chlorosome, a flattened ellipsoidal organelles appressed to the cytoplasmic face of the cell membrane which serve as common antennas for several reaction centers [5-7]. Main light-harvesting pigment in chlorosome is bacteriochlorophyll c, beside this carotene alpha, beta and bateriochlorophyll a are also present in chlorosome [1-4]. Autotrophic CO_2_ fixation is carried by 3-hydroxypropionate cycle and in each turn of the cycle two molecules of bicarbonate is fixed into one molecule of glyoxylate [8-13]. This thermophilic bacterium encodes some heat stable enzymes such as enolase, metalloprotease I, malto-tiose and malto-tetraose that produce amylase which has role in food, pharmaceutical, and fine-chemicals industries [7, 14-16]. *Chloroflexus aurantiacus *have been isolated from different hot springs (50-60°C) throughout the world. The complete genome of *Chloroflexus aurantiacus *J-10-fl was sequenced and published in 2011 which was isolated from Hakone hot spring (52-60°C) area in Japan. The genome sequence analysis revealed a total of 3853 proteins but 785 (~20%) of these proteins are uncharacterized or hypothetical proteins (HPs), the proteins which are predicted from nucleic acid sequences and for which there is no experimental evidences regarding their functions [7, 17].

Protein sequence databases contain huge number of HPs that must be annotated. Annotation of hypothetical proteins play crucial role to understand the biological role, gene regulation, new target identification, function and pathway analysis [18-20]. Characterizations of large number of proteins are not possible using *in-vitro* lab experimentations so bioinformatics approaches can play significant role in functional annotation. These approaches has been successfully implemented for many bacterial species such as *Vibrio cholera* [21], *Neisseria gonorrhoeae* [22], *Clostridium difficile* [23], *Staphylococcus aureus *[24], *Haemophilus influenzaeRd *KW20 [25], *Leishmania donovani* [26], *Shigella *flexneri [27], *Helicobacter pylori* [28] and *Exiguobacterium antarcticum *B7 [29]. Thus here in this work we have used different bioinformatics functional annotation tools and databases to characterize or annotate HPs of *Chloroflexus aurantiacus *J-10-fl.

## MATERIALS AND METHODS

**Data retrieval: **In this study, we retrieved 785 HPs of *Chloroflexus aurantiacs *J-10-fl from UniProtKB [30] (https://www.uniprot.org/) for annotation. All HPs sequences were downloaded in FASTA format for functional annotation.

**Prediction of protein domains or families: **HPs were analyzed to find out functionally important features such as domains or protein families. Domains are structurally and functionally conserved regions in the proteins responsible for a particular function or interaction whereas protein family helps in classifying the proteins based on associated functions or sequence similarities. Therefore searching domains or protein families of HPs is the initial step to figure out the functional role. It provides overall functionality of the proteins thereby act as pivotal for providing an idea about the function of the uncharacterized protein [31]. For the identification of these structurally and functionally important regions various tools and databases like CDD (Conserved Domain Database) (https://www.ncbi.nlm.nih.gov/ Structure/cdd/wrpsb.cgi) [32], Pfam (Protein Family) (https://pfam.xfam.org/) [33], InterPro (https://www.ebi.ac.uk/interpro/) [34], SVMprot (Support Vector Machine Protein functional family prediction) (http://bidd2.nus.edu.sg/cgi-bin/svm-prot/svmprot.cgi) [35], SMART (Simple Modular Architecture Research Tool) (http://smart.embl-heidelberg.de/) [36] and CATH (Class Architecture Topology Homology) (https://www.cathdb.info/) [37] were used in our studies with default parameters. After analysis of all HPs by domain or protein family prediction tools only those HPs were considered for further annotation whose function is predicted by five or more tools. These filtered HPs were then termed as HC-HPs (high confidence – hypothetical proteins).

**Prediction of physiochemical parameters and sequence homology: **ProtParam (https:// web.expasy.org/protparam/) [38] was used for the determination of physiochemical parameters such as molecular weight, instability index, and aliphatic index, grand average of hydropathicity (GRAVY), extinction coefficient, and theoretical pI of HC-HPs. The homology based annotation by sequence comparison helps in predicting the function of uncharacterized proteins. Therefore, the comparative sequence alignment of all HC-HPs with already annotated protein database sequences was performed by BLASTP (https://blast.ncbi.nlm.nih.gov/Blast.cgi) using default parameters [39]. 

**Prediction of sub-cellular localization: **Sub-cellular localization prediction provides information about the compartmentalization of proteins in cell organelles. Additionally, a protein cellular location prediction helps in functional annotation. Hence, to predict sub-cellular location of HC-HPs we used different sub-cellular prediction web-servers: PSORTb 3.0.2 (https://www.psort.org/psortb/) [40, 41], PSLpred (Prediction of bacterial Subcellular Localization) (http://crdd.osdd.net/raghava/pslpred/) [42] and CELLO (subcellular Localization) (http://cello.life.nctu.edu.tw/) [43]. For the presence of signal peptide and secretary proteins, SignalP 5.0 (http://www.cbs.dtu.dk/services/SignalP/) [44] and SecretomeP 2.0 server (http://www.cbs.dtu.dk/services/SecretomeP/) [45] was used. It verify the location of cleavage site by classical pathway and non-classical secretion i.e. inactivation of Sec-dependent secretion pathways respectively [46, 47].

**Transmembrane helices and topology prediction:** To predict the topology i.e. membrane or soluble nature of HC-HPs we used three web-servers named TMHMM (Hidden Markov Model for Transmembrane Helices) (http://www.cbs.dtu.dk/services/TMHMM/) [48], HMMTOP (Hidden Markov Model for TOpology Prediction) (http://www.enzim.hu/hmmtop/) [49] and SOSUI (http://harrier.nagahama-i-bio.ac.jp/sosui/) [50]. TMHMM and HMMTOP predict number of transmembrane regions whereas SOSUI classify proteins in to soluble or membrane protein.

**Figure 1:**
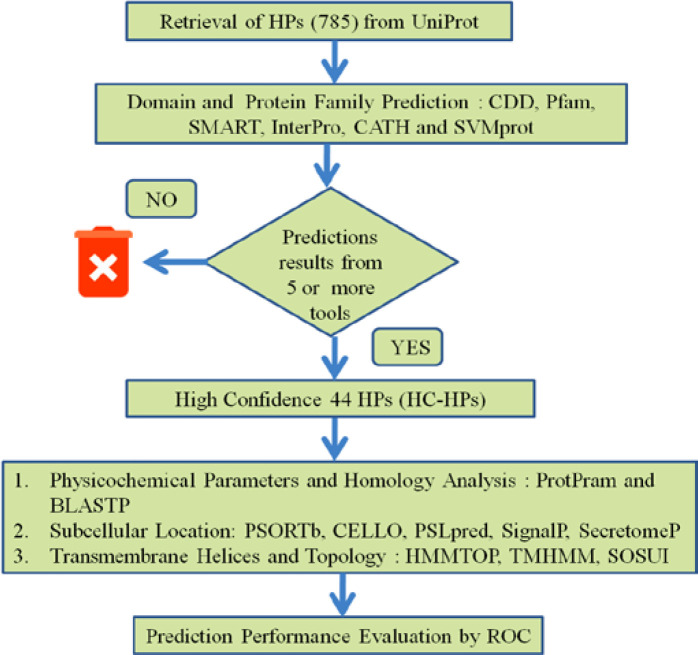
Workflow of Methodology

**Performance assessment: **To validate the prediction outcome, receiver operating characteristic (ROC) was performed. ROC is widely used method to compare the accuracy of prediction models [25, 27, 30, 51]. We have used six levels to evaluate diagnostics efficacy in which two binary numerals ‘0’ or ‘1’ classify the prediction, where ‘1’ denotes a true positive and ‘0’ denotes a true negative and 2, 3, 4 and 5 integers were used for confidence rating. A web based server (http://www.rad.jhmi.edu/jeng/javarad/roc/JROCFITi.html) [52] is used to verify the domain prediction accuracy of 44 HC-HPs. The dataset used for ROC analysis is listed in Supplementary Table S2. For this purpose, we have randomly selected 25 proteins of *Chloroflexus aurantiacus *J-10-f1 with known function from UniProt. These proteins functions are predicted by same tools that we have selected for in our work. After the assigning the confidence score to each protein in the form of binary numerals as mentioned above the data was submitted to ROC analysis in format ‘1’ option of the webserver. The statistical parameters of prediction assessment such as sensitivity, specificity and the ROC area was noted The detailed flowchart of methodology is described in Figure 1.

## RESULTS AND DISCUSSION

Over the past two decades there is generation of large amount of genome sequencing data that is conceptually translated in to protein sequences. This large amount of protein sequence data archived in databases like UniProt both in reviewed or unreviewed form. With the beginning of genomics or proteomics era there is simultaneous increase in development of bioinformatics prediction algorithms or tools. These bioinformatics prediction tools are being utilized successfully to annotate or characterize the large amount of gene or protein sequence data [53].

**Figure 2:**
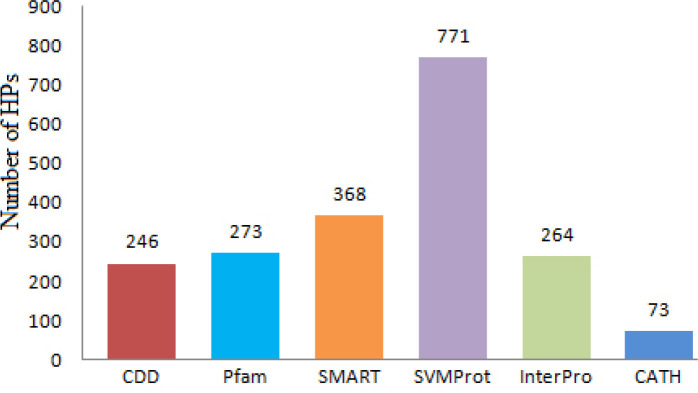
Number of HPs predicted by each tool

Here in this work we have retrieved 785 HPs from UniProt database which is 20% of Chloroflexus aurantiacus J-10-fl’s total proteins. Initial functional annotation analysis was done by domain or protein family prediction tools and databases like SMART, CDD, Pfam, SVMProt, CATH and InterPro to find conserved domains. The analysis result of domain or characterization is listed in Supplementary Table S1**. **Total numbers of annotation made by each tool or database are mention in Fig. 2 which indicated that SVMProt annotated highest number proteins and CATH predicted least annotation. 

HPs annotated by five or more tools were filtered and designated as HC-HPs. The downstream analysis was done using these high confidence hypothetical proteins, HC-HPs. We have filtered 44 HCPs (Table 1) on the basis of prediction results of five or more tools and assigned functions which were retrieved from InterPro which is the consortium of databases. InterPro annotation results are derived from 14 different specialist member databases which uses diagnostic models like HMMs, other forms of profiles, position-specific scoring matrices, and regular expressions, collectively known as signatures, against which protein sequences is searched to assign potential functions. Signature of member databases undergoes manual annotation and integration process [31, 44].

**Table 1:** Functional Classification to the HC-HPs

**Uniprot A/C**	**InterPro Classification**
A9WC42	Alpha helical ferredoxin
A9WC74	BisanhydrobacterioruberinhydrataseCruF like
A9WC89	Beta lactamase hydrolase like
A9WCX1	P loop containing nucleoside triphosphate hydrolase
A9WD08	YwiC like protein
A9WDN5	Pectin lyase fold
A9WE66	ABC 2 family transporter protein
A9WE95	Stage II sporulation protein M like
A9WEQ6	Carbohydrate binding domain containing protein Cthe_2159
A9WAF1	O antigen ligase related
A9WAV7	LPPG:FO 2 phospho L lactate transferaseCofD/UPF0052
A9WBQ0	Glycoside hydrolase family 30
A9WBU0	Glycoside hydrolase superfamily
A9WCD7	Peptidase C11, clostripain
A9WCF4	PDZ superfamily
A9WD37	Putative zincin peptidase
A9WD59	4Fe4S_Fe-S-bd, NarG like superfamily,Cys_rich_dom,
A9WE09	Cellulose synthase BcsB, bacterial
A9WEE4	tRNAthreonylcarbamoyl adenosine modification protein TsaE
A9WEL0	Small GTPase superfamily, ARF/SAR type
A9WEX4	Immunoglobulin like fold
A9WF32	Glycolate oxidase, iron sulphur subunit
A9WFR2	Epimerase family protein SDR39U1
A9WGG2	Rhamnose/fucosemutarotase
A9WDH7	Photosynthetic complex assembly protein 2, putative
A9WDG5	P loop containing nucleoside triphosphate hydrolase
A9WCP0	FAD binding, type PCMH, subdomain 2
A9WCL1	P loop containing nucleoside triphosphate hydrolase
A9WBY1	S adenosyl L methionine dependent methyltransferase
A9WB90	Immunity protein 26
A9WKM2	DoxX family
A9WA42	Flagellar filament outer layer protein FlaA
A9WGV0	EamA domain
A9WG43	Peptidase S49, serine peptidase prokaryotes
A9WHY4	Alpha galactosidase, NEW3 domain
A9WIW6	Six bladed beta propeller, TolB like
A9WHZ1	Phosphate regulon sensor protein PhoR
A9WJM5	RmlC like jelly roll fold
A9WJF9	Transmembrane protein TqsA like
A9WHC9	Restriction endonuclease type II like
A9WGM3	Lamin tail domain superfamily
A9WAT0	1,4 alpha glucan branching enzyme MT3115 like
A9WAN6	Transmembrane protein TqsA like
A9WJJ6	CCB3/YggT

Domain and BLAST analysis characterized HC-HPs role as the member of photosynthetic complex. Four HC-HPs with accession number: A9WC42, A9WC74, A9WD59 and A9WDH7 were found to the members of photosynthesis complex. For instance, A9WC42 showed 88.96% identity with (Fe-S)-binding protein of *Chloroflexus aggregans* and A9WC74 was 88.77% identical to carotenoid biosynthesis protein of *Chloroflexus islandicus. *The other two members A9WD59 and A9WDH7 were 95.64% and 61.67% identical to 4Fe-4S dicluster domain-containing protein of *Chloroflexus sp. *MS-G and photosynthetic complex assembly protein of *Oscillochloris trichoides* DG6 respectively.

It was further found that six HC-HPs with accession number A9WEQ6, A9WE09, A9WAF1, A9WAT0, A9WBQ0 and A9WBU0 were involved in carbohydrate metabolism processes. Protein A9WEQ6 contain carbohydrate binding domain containing protein which is member of novel family of cellulose and/or acid-sugar that share unique right-handed parallel β-helix protein structural similarities with polysaccharide lyases from *Clostridium thermocellum* and has great application as biofuel production [54, 55]. Whereas, HC-HP A9WE09 is a membrane associated bacterial cellulose synthase (Bcs)B subunit [56, 57]. Bacterial cellulose fulfills industrial and biomedical needs due to its high purity, high degree of polymerization, high crystalline, high water content as well as high mechanical stability. [58]. A9WAF1 is involved in synthesis of O-antigen (lipopolysaccharides) and A9WAT0 act as hydrolase that catalyzes the formation of branch points in glycogen and amylopectin of hyperthermophilic archaeon of *Thermococcus kodakaraensis* KOD1 [59, 60]. A9WBQ0 and A9WBU0 is member of glycoside hydrolase that hydrolyse the glycosidic bond between two or more carbohydrates, or between a carbohydrate and a non-carbohydrate moiety [61, 62].

Furthermore, physicochemical properties like molecular weight, isoelectric point, aliphatic index and GRAVY score were calculated by ProtParam (Table 2). The aliphatic index representing the volume occupied by the aliphatic residues (Ala, Val, Leu, and Ile) which consequently indicate the increased thermal stability for globular proteins thereby a greater aliphatic index value represents higher thermally stable protein. Greater aliphatic indices and greater hydrophobicity indices show a positive inclination towards the thermo stability of the proteins. The GRAVY value representing the protein water interaction, the proteins having low gravy score interact better with water and higher GRAVY score indicate higher hydrophobicity [39]. On the basis of physiochemical parameter we have filtered three thermostable proteins with accession number A9WEE4, A9WEL0 and A9WDG5. All three HC-HPs have molecular weight less than 30kD, theoretical pI range from 4.5-10.0, aliphatic index value >90 and low GRAVY scores which is comparable with other thermostable proteins [30]. 

**Table 2:** Physicochemical Parameters Characterization of HC-HPs by ProtParam

**UniProt A/C**	**No. of amino acids**	**MW**	**PI**	**EC**	**IN**	**C**	**AI**	**GRAVY**
A9WC42	511	57267.99	5.92	53830	44.96	unstable	93.72	-0.114
A9WC50	303	32839.06	5.09	26470	48.41	unstable	116.2	0.275
A9WC89	169	19165.43	4.96	31065	45.57	unstable	75.21	-0.38
A9WCX1	503	55013.84	6.39	93515	44.17	unstable	99.11	-0.098
A9WD08	270	29275.23	11.49	78950	42.24	unstable	133.78	0.883
A9WDN5	1093	111934.68	5.37	60975	26.53	stable	94.52	0
A9WE66	358	39514.58	9.99	68410	45.19	unstable	131.79	0.847
A9WE95	318	34766.78	9.29	46535	36.2	stable	126.73	0.527
A9WEQ6	575	57404.8	4	22015	20.02	stable	95.06	0.208
A9WAF1	511	58050.12	10.05	125820	45.72	unstable	121.14	0.683
A9WAV7	391	43267.39	6.16	37485	40.53	unstable	119.67	0.225
A9WBQ0	461	50401.05	4.59	92485	42.22	unstable	88.29	0.005
A9WCD7	907	97634.61	4.58	99615	39.74	stable	98.29	0.009
A9WCF4	511	57752.24	5.23	55850	48.52	unstable	95.62	-0.166
A9WD37	191	21004.74	6.96	23950	33.75	stable	126.6	0.542
A9WD59	719	80830.12	5.9	113425	48.75	unstable	94.87	-0.08
A9WE09	687	73815.89	5.53	94545	42.01	unstable	108.37	0.158
A9WEE4	194	21923.2	7.17	26930	27.56	stable	100.62	-0.157
A9WEL0	195	22046.74	9.51	14440	37.55	stable	98.92	-0.044
A9WEX4	735	78497.61	4.3	86430	24.92	stable	70.76	-0.151
A9WF32	466	50442.28	8.62	33890	41.79	unstable	90.94	-0.044
A9WFR2	317	34356.5	7.76	53525	37.01	stable	97.29	-0.003
A9WGG2	107	12495.26	5.11	23950	57.52	unstable	75.7	-0.324
A9WDH7	254	28099.11	8.63	84910	49.37	unstable	122.28	0.655
A9WDG5	192	21106.34	6.28	17990	41.23	unstable	98.07	-0.016
A9WCP0	433	48100.26	4.65	38390	38.63	stable	116.84	0.134
A9WCL1	544	63433.76	9.15	134885	46.62	unstable	89.21	-0.393
A9WBY1	825	89589	8.91	126740	35.24	stable	115.94	0.531
A9WBA3	101	11474.1	4.94	22460	23.25	stable	77.23	-0.231
A9WB90	351	40008.15	7.77	90870	39.52	stable	69.2	-0.671
A9WKM2	195	21643.38	8.86	61420	25.63	stable	99.59	0.461
A9WA42	908	99720.92	5.36	183120	36.64	stable	79.82	-0.28
A9WGV0	297	31049.12	9.92	42400	25.24	stable	145.29	1.059
A9WG43	274	30811.93	5.85	19940	60.27	unstable	112.85	0.027
A9WHY4	389	41527.4	5.26	26930	37.89	stable	97.25	0.064
A9WIW6	678	71207.78	4.59	80245	37.73	stable	91.43	0.102
A9WHZ1	286	31566.03	8.46	32220	43.84	unstable	120.38	0.313
A9WJM5	613	68498.39	6.16	53830	44.96	unstable	93.72	-0.114
A9WJF9	432	46351.65	5.59	56380	51.47	unstable	128.96	0.743
A9WHC9	377	42791.5	5.22	42400	48.24	unstable	76.87	-0.76
A9WGM3	824	86307.05	4.78	136025	36.45	stable	86.04	-0.08
A9WAT0	485	55746.41	6.08	99935	49.25	unstable	98.6	-0.278
A9WAN6	386	42700.88	8.75	66350	38.79	stable	142.23	0.857
A9WJJ6	85	9584.54	6.53	6990	57.44	unstable	133.06	0.976

MW: Moleular Weight, PI: Isoelectric Point, EC: Extinction Coefficient, IN: Instability index, C: Classificatio, AI: Alphabatic index, GRAVY: Grand average of hydropathicity

Additionally, these thermostable HC-HPs show significant homology and sequence coverage with database archived thermophilic proteins that provide resistance to bacteria at high temperature. A9WDG5 showed 95% of query coverage and 39.36 percent identity with AAA+ family ATPases of* Thermosporothrix hazakensis* that grows at 31°C to 58°C , with the pH range of 5.4 to 8.7 [63-64]. While A9WEE4 showed 53.18 percentage identity and 85% coverage with tRNA (adenosine (37)-N6)-threonylcarbamoyltransferase complex ATPase subunit type 1 TsaE enzymes of thermophilic bacteria Roseiflexus that found at 50°C at pH range of 7.5 to 8.0 [65, 66]. A9WEL0 showed 62-65% identity and 100% query coverage with GTPase domain-containing protein of *Thermosporothrix hazakensis* which were isolated at 55°C, pH 8.5 from thermal treated sewage sludge in Germany [67].

Subcellular localization, signal peptides and transmembrane analysis of HPs are mentioned in Supplementary Table S3. These features are crucial to unveil new target for drug discovery, secretory pathways, cell–cell signaling, transport of ions and solutes across the membrane [43]. Web based calculator for ROC, calculate the accuracy, sensitivity, specificity and the ROC area of the functional prediction [53]. This statistical estimation method is extensively used for analyzing and comparing the accuracy of predicted function. The average accuracy provided by ROC for used pipeline was 92.6% which indicated the high reliability of the set of bioinformatics tools used in our study (Table 3).

**Table 3:** Sensitivity, specificity, accuracy and ROC area of the tools used for the annotation of functionally known 25 proteins from *Chloroflexus aurantiacus *J-10-f1 by ROC

**Software**	**Sensitivity**	**Specificity**	**Accuracy**	**ROC Area**
CDD	95.80%	100%	96%	0.979
Pfam	100.00%	100%	100%	1
SMART	77.30%	100%	80.00%	0.886
SVMProt	85.70%	100%	88%	0.929
InterPro	95.80%	100%	96%	0.979
CATH	95.70%	100%	96%	0.978
**Average**	**91.71%**	**100%**	**92.66%**	**0.958**

The bioinformatics pipeline used by us provides comprehensive annotation of uncharacterized proteins of *Chloroflexus aurantiacs *J-10-f1. Some HC-HPs were found to have thermophilic nature and thus can protect bacteria at high temperature. Beside this a few HC-HSPs are involved in carbohydrate metabolism. Thus these annotated proteins can be of significance for industrial as well as in biomedical needs. Our findings may further be verified experimentally.

## Supplementary Materials

Supplementary Material 1

Supplementary Material 2

Supplementary Material 3
