# Supplementary material for: Deciphering the functional role of hypothetical proteins from Chloroflexus aurantiacs J-10-f1 using bioinformatics approach
Source: Mol Biol Res Commun. 2020 Sep;9(3):129–39. doi: 10.22099/mbrc.2020.36894.1495 (PMC7727763; doi:10.22099/mbrc.2020.36894.1495)
Supplement: Supplementary Material 1 [file mbrc-9-129-s001.pdf]

Table S1 : Annotation results for the 785 hypothetical proteins after analysis with CDD, Pfam, SMART, SVMprot, InterPro and CATH and filtration of HPs result by 5 or more tools and called them HC-HPs (High Confidence HPs)

S.NO.	Uniprot A/C	CDD	Pfam	SMART	SVMprot	InterPro	CATH	HC-HPs
1	A9WBD2	DUF309	DUF309	No	EC5.5	Protein of	TTHA0068-like	NO
2	A9WBD5	No Result	No Result	Transme	TC9.A	No Result	No Result	NO
3	A9WBD6	No Result	No Result	No	All lipid binding	No Result	No Result	NO
4	A9WBE0	No Result	No Result	Transme	7	No Result	No Result	NO
5	A9WBE4	DUF1405	DUF1405	Transme	TC2.A	DUF1405	No Result	NO
6	A9WBF8	No Result	No Result	No	No Result	No Result	No Result	NO
7	A9WBF9	No Result	No Result	No	Manganese	No Result	No Result	NO
8	A9WBG1	MreD super	No Result	transmem	TC3.A.5 Type II	No Result	No Result	NO
9	A9WBH1	DUF4380 super	No Result	No	Iron binding	No Result	No Result	NO
10	A9WBH4	No Result	No Result	No	Zinc binding	No Result	No Result	NO
11	A9WBH5	Atrophin 1 super	No Result	No	Zinc binding	No Result	No Result	NO
12	A9WBH9	No Result	No Result	No	Metal binding	No Result	No Result	NO
13	A9WBI0	No Result	No Result	No	Zinc binding	No Result	No Result	NO
14	A9WBI3	No Result	No Result	transmem	All lipid binding	No Result	No Result	NO
15	A9WBI6	No Result	No Result	No	Iron binding	No Result	No Result	NO
16	A9WBI9	SRPBCC	Polyketide_cyc2	No	EC3.1	Polyketidecyclase/	No Result	NO
17	A9WBJ0	U1snRNP70_N	No Result	transmem	TC1.E	No Result	No Result	NO
18	A9WBJ5	No Result	No Result	No	Iron binding	No Result	No Result	NO
19	A9WBK1	NYN_YacP	NYN_YacP	No	No Result	Protein of	No Result	NO
20	A9WC22	No Result	No Result	Signal	All lipid binding	No Result	No Result	NO
21	A9WC23	CSP_CDS	No Result	Transme	All lipid binding	No Result	Cold shock protein cspA	NO
22	A9WC37	No Result	No Result	No	EC2.1	No Result	No Result	NO
23	A9WC38	No Result	No Result	No	Magnesium	No Result	No Result	NO
24	A9WC42	GlpC	Fer4_8,7,9,17,CC	No	Zinc binding	Alpha helical	Heterodisulfide reductase	YES
25	A9WC43	No Result	No Result	transmem	EC2.4	No Result	No Result	NO
26	A9WC46	No Result	No Result	No	All lipid binding	No Result	No Result	NO
27	A9WC50	DUF4349	DUF4349	transmem	calcium-binding	Domain of	No Result	NO
28	A9WC59	No Result	No Result	No	EC3.1	No Result	No Result	NO
29	A9WC60	No Result	No Result	No	EC1.1	No Result	No Result	NO
30	A9WC72	No Result	SURF1	No	EC3.1	No Result	No Result	NO
31	A9WC74	Caroten_synth	Caroten_synth	Transme	EC1.10	Bis	No Result	YES
32	A9WC76	No Result	No Result	No	Zinc binding	No Result	No Result	NO
33	A9WC78	COG3355	No Result	No	Iron binding	Wingedhelix	No Result	NO
34	A9WC83	No Result	AAA_assoc_C	No	Zinc binding	C terminalAAA	ABC transporter ATP	NO
35	A9WC87	DrsE_2	DrsE,_2	No	All lipid binding	DsrE2 like family	No Result	NO
36	A9WC89	PTPc superfamily	DUF442	No	Iron binding	Beta lactamase	Beta lactamase	YES
37	A9WC97	No Result	No Result	Transme	Metal binding	No Result	No Result	NO
38	A9WCA2	DUF2342_F420	Zincin_2	No	EC3.6	Zinicin like	No Result	NO
39	A9WCT5	MutL	MutL	No	Zinc binding	ProteinMutL	No Result	NO
40	A9WCT6	No Result	No Result	Transme	Metal binding	No Result	No Result	NO
41	A9WCT7	DNA_pol3_gamm	No Result	No	Zinc binding	No Result	No Result	NO
42	A9WCT8	No Result	No Result	Transme	TC2.A	No Result	No Result	NO
43	A9WCT9	No Result	No Result	Transme	Zinc binding	No Result	No Result	NO
44	A9WCU0	No Result	No Result	No	TC2.A	No Result	No Result	NO
45	A9WCU1	SRPBCC_Smu44	Polyketide_cyc2	No	Manganese	Polyketidecyclase/	No Result	NO
46	A9WCW4	No Result	No Result	Transme	Sodium binding	No Result	No Result	NO
47	A9WCW7	DUF3987	DUF3987	No	Zinc binding	Protein of	No Result	NO
48	A9WCW8	No Result	No Result	No	EC2.5	No Result	No Result	NO
49	A9WCW9	tRNAN synt_1g	No Result	No	EC2.4	No Result	No Result	NO
50	A9WCX0	No Result	No Result	transmem	EC2.7	No Result	No Result	NO
51	A9WCX1	AAA,P	No Result	Transme	Metal binding	P loop containing	DNA translocase FtsK 1	YES
52	A9WCX2	No Result	No Result	No	EC2.4	No Result	No Result	NO
53	A9WCX6	GlyGly_RbtA	No Result	Transme	All lipid binding	Pectinlyasefold/vir	No Result	NO
54	A9WCX7	DUF3592	DUF3592	No	TC2.A	Protein of	No Result	NO
55	A9WCX8	DNA_pol3_delta2	No Result	signal	All lipid binding	No Result	No Result	NO
56	A9WCX9	No Result	No Result	Transme	TC2.A	No Result	No Result	NO
57	A9WCY1	No Result	No Result	Transme	TC2.A.1 Major	No Result	No Result	NO
58	A9WCY2	No Result	No Result	signal	No Result	No Result	No Result	NO
59	A9WCY7	No Result	No Result	transmem	TC1.E	No Result	No Result	NO
60	A9WCY8	MerR_2	MerR_2	No	No Result	No Result	No Result	NO
61	A9WCZ1	eRF1_3	No Result	No	EC2.7	Bacterialarchaeo	No Result	NO
62	A9WCZ2	DUF2905	DUF2905	No	TC3.A Primary	Proteinofunknownf	No Result	NO

63	A9WCZ8	No Result	No Result	No	All lipid binding	No Result	No Result	NO
64	A9WCZ9	No Result	No Result	No	Nuclear	No Result	Anti Muellerian hormone	NO
65	A9WD01	No Result	ABC_cobalt	transmem	7	ABCtypethiamin	No Result	NO
66	A9WD08	YwiC	YwiC	transmem	TC2.A	YwiC like protein	No Result	YES
67	A9WD18	No Result	No Result	No	TC3.A.5 Type II	No Result	No Result	NO
68	A9WD27	SMC_N	No Result	coiled	All lipid binding	No Result	No Result	NO
69	A9WD29	No Result	No Result	No	Iron binding	No Result	No Result	NO
70	A9WDL0	No Result	No Result	No	EC2.1	No Result	No Result	NO
71	A9WDL6	No Result	No Result	No	Iron binding	No Result	No Result	NO
72	A9WDM7	rpmI	Ribosomal_L35p	No	TC3.A.5 Type II	RibosomalproteinL	No Result	NO
73	A9WDN1	No Result	No Result	No	Copper binding	No Result	No Result	NO
74	A9WDN2	RhaT	EamA	No	TC2.A	No Result	No Result	NO
75	A9WDN4	No Result	No Result	signal	EC5.3	No Result	No Result	NO
76	A9WDN5	Beta_helix,CARD	NPCBM_assoc	PbH1	Outer membrane	Pectin lyase fold	No Result	YES
77	A9WDP5	No Result	No Result	transmem	EC3.6	No Result	No Result	NO
78	A9WDP8	No Result	No Result	coiled	Iron binding	No Result	No Result	NO
79	A9WDP9	No Result	No Result	transmem	All lipid binding	No Result	No Result	NO
80	A9WDQ5	No Result	No Result	No	All DNA	No Result	No Result	NO
81	A9WDR2	DUF169	No Result	No	EC3.4	Protein of	No Result	NO
82	A9WDS3	Phage_holin_4_2	Phage_holin_4_2	No	TC1.E	Mycobacterial 4	No Result	NO
83	A9WDT1	ABC2_membrane	ABC2_membrane	No	TC3.A.15 The	Protein of	No Result	NO
84	A9WDT3	No Result	No Result	No	All lipid binding	No Result	No Result	NO
85	A9WDT5	PHA03102	No Result	coiled	All DNA	Chaperone J	No Result	NO
86	A9WE33	SapB superfamily	No Result	No	Zinc binding	No Result	No Result	NO
87	A9WE41	No Result	No Result	transmem	TC2.A	Six bladedbeta	No Result	NO
88	A9WE43	Ferritin_like	No Result	transmem	Metal binding	No Result	No Result	NO
89	A9WE46	DUF4129	No Result	No	rRNA binding	No Result	No Result	NO
90	A9WE64	No Result	No Result	transmem	TC9.B	No Result	No Result	NO
91	A9WE66	ABC2_membrane	ABC2_membrane	transmem	TC4.A Group	ABC 2 family	No Result	YES
92	A9WE68	GATase1_like	No Result	transmem	EC2.5	No Result	No Result	NO
93	A9WE91	No Result	No Result	transmem	7	No Result	No Result	NO
94	A9WE95	SpoIIM	SpoIIM	transmem	EC2.7	Stage II sporulation	No Result	YES
95	A9WE96	PMT_2	PMT_2	transmem	Zinc binding	Protein of	No Result	NO
96	A9WE97	DUF192	DUF192	No	EC2.4	Proteinofunknownf	No Result	NO
97	A9WEA3	UDG_like	No Result	No	EC2.4	No Result	No Result	NO
98	A9WEA5	PKc_like	No Result	No	EC1.1	No Result	No Result	NO
99	A9WEA6	DUF3037	DUF3037	No	TC2.A	Proteinofunknownf	No Result	NO
100	A9WEA7	No Result	No Result	No	EC2.4	No Result	No Result	NO
101	A9WEB1	No Result	No Result	No	EC2.7	No Result	No Result	NO
102	A9WEB3	Sortilin Vps10	No Result	No	All lipid binding	No Result	Probable oligoxyloglucan	NO
103	A9WEC2	LPG_synthase_T	LPG_synthase_T	No	TC2.A	Lysylphosphatidylg	No Result	NO
104	A9WEC5	RtcB	RtcB	No	Zinc binding	tRNA splicing	No Result	NO
105	A9WEN4	No Result	No Result	No	EC2.1	No Result	No Result	NO
106	A9WEN5	No Result	No Result	transmem	TC1.C	No Result	No Result	NO
107	A9WEN6	DUF3179	DUF3179	signal	EC3.2	Protein of	No Result	NO
108	A9WEP4	No Result	No Result	No	TC3.A.15 The	No Result	No Result	NO
109	A9WEP5	Csx1_IIIU	No Result	No	EC3.1	No Result	No Result	NO
110	A9WEQ2	No Result	No Result	transmem	All lipid binding	Cupredoxin	No Result	NO
111	A9WEQ4	No Result	PCuAC	signal	EC2.7	Copper chaperone	No Result	NO
112	A9WEQ5	No Result	No Result	transmem	TC3.A.5 Type II	No Result	No Result	NO
113	A9WEQ6	PCuAC,Cthe_215	Cthe_2159	signal	All lipid binding	Carbohydrate	No Result	YES
114	A9WER6	TthCsoR	Trns_repr_metal	No	rRNA binding	Metal	No Result	NO
115	A9WES3	No Result	No Result	transmem	Copper binding	No Result	No Result	NO
116	A9WES4	DUF507	DUF507	No	EC3.6	Proteinofunknownf	No Result	NO
117	A9WES6	No Result	No Result	transmem	EC2.4	No Result	No Result	NO
118	A9WET1	No Result	No Result	transmem	All lipid binding	No Result	No Result	NO
119	A9WET3	No Result	No Result	No	EC3.1	No Result	No Result	NO
120	A9WET5	No Result	No Result	transmem	EC2.4	No Result	No Result	NO
121	A9WET6	No Result	No Result	transmem	Metal binding	No Result	No Result	NO
122	A9WEU0	No Result	No Result	No	All lipid N	No Result	No Result	NO
123	A9WEU5	No Result	No Result	No	EC2.7	Proteinkinase	No Result	NO
124	A9WEU6	No Result	No Result	transmem	All lipid binding	No Result	No Result	NO
125	A9WEV7	No Result	No Result	transmem	All lipid binding	No Result	No Result	NO
126	A9WEV8	No Result	No Result	transmem	Iron binding	No Result	No Result	NO
127	A9WEV9	No Result	DUF123	No	EC2.7	Proteinofunknownf	No Result	NO
128	A9WFE0	No Result	No Result	signal	EC2.4	No Result	Outer membrane adhesin	NO

129	A9WFE1	No Result	No Result	transmem	All lipid binding	No Result	No Result	NO
130	A9WFE2	No Result	No Result	signal	All lipid binding	Sialidasesuperfami	Outer membrane adhesin	NO
131	A9WFE3	No Result	No Result	signal	EC2.4	No Result	No Result	NO
132	A9WFE8	No Result	No Result	No	EC2.7	Wingedhelix	No Result	NO
133	A9WFF5	No Result	No Result	HNHc	Zinc binding	No Result	No Result	NO
134	A9WFF6	No Result	No Result	transmem	G protein	No Result	No Result	NO
135	A9WFG1	No Result	No Result	No	Iron binding	No Result	No Result	NO
136	A9WFG4	No Result	No Result	No	EC2.4	No Result	No Result	NO
137	A9WFG9	No Result	DUF1385	No	EC2.4	Proteinofunknownf	No Result	NO
138	A9WFH0	No Result	No Result	transmem	EC4.6 Lyases	No Result	No Result	NO
139	A9WFH1	No Result	No Result	signal	All lipid binding	No Result	No Result	NO
140	A9WFI3	No Result	No Result	transmem	EC3.6	No Result	No Result	NO
141	A9WFJ5	No Result	No Result	transmem	TC2.A	No Result	No Result	NO
142	A9WFL6	No Result	DUF87	No	EC3.6	No Result	No Result	NO
143	A9WFL9	No Result	No Result	signal	Zinc binding	No Result	No Result	NO
144	A9WG53	DUF4838,Glyco_	DUF4838	No	Zinc binding	Proteinofunknownf	No Result	NO
145	A9WG55	No Result	No Result	transmem	Chlorophyll	No Result	No Result	NO
146	A9WG56	No Result	No Result	transmem	Zinc binding	No Result	No Result	NO
147	A9WG71	No Result	No Result	transmem	EC2.4	No Result	No Result	NO
148	A9WG79	No Result	No Result	coiled	rRNA binding	No Result	No Result	NO
149	A9WG83	No Result	No Result	No	All lipid binding	No Result	No Result	NO
150	A9WG84	No Result	No Result	No	All lipid binding	No Result	No Result	NO
151	A9WG85	No Result	No Result	coiled	EC2.4	Tetratricopeptide	No Result	NO
152	A9WG91	No Result	No Result	CDC37_	EC2.5	Tetratricopeptide	No Result	NO
153	A9WG93	No Result	EamA	No	TC2.A	EamAdomain	No Result	NO
154	A9WG99	No Result	No Result	transmem	EC3.6	No Result	No Result	NO
155	A9WGA0	No Result	No Result	transmem	TC4.A Group	No Result	No Result	NO
156	A9WGA1	No Result	No Result	No	rRNA binding	No Result	No Result	NO
157	A9WGA6	No Result	No Result	No	All lipid binding	No Result	No Result	NO
158	A9WGB2	No Result	Protoglobin	No	All lipid binding	Protoglobin	No Result	NO
159	A9WGC4	No Result	No Result	No	rRNA binding	No Result	No Result	NO
160	A9WGC5	No Result	PDDEXK_1	No	Metal binding	No Result	No Result	NO
161	A9WGC6	No Result	No Result	transmem	All lipid binding	No Result	No Result	NO
162	A9WGD4	No Result	FtsX	No	All DNA	No Result	No Result	NO
163	A9WGW4	No Result	DUF4230	transmem	TC2.A	ABCtransporterper	No Result	NO
164	A9WGW9	No Result	CCG	No	Zinc binding	Cysteinerichdomai	No Result	NO
165	A9WGX2	No Result	No Result	No	EC3.1	No Result	No Result	NO
166	A9WGY8	No Result	No Result	No	Zinc binding	No Result	Serine/threonine protein	NO
167	A9WH00	No Result	DUF4230	transmem	EC2.4	No Result	No Result	NO
168	A9WH01	No Result	DUF4230	Signal	All lipid binding	Proteinofunknownf	No Result	NO
169	A9WH15	No Result	No Result	transmem	EC1.15	No Result	No Result	NO
170	A9WH20	No Result	No Result	transmem	Manganese	No Result	No Result	NO
171	A9WH21	No Result	No Result	coiled	All DNA	No Result	No Result	NO
172	A9WH26	No Result	No Result	No	EC3.1	No Result	No Result	NO
173	A9WH30	No Result	No Result	signal	EC2.7	No Result	RING finger protein nhl 1	NO
174	A9WH33	No Result	No Result	No	All lipid binding	No Result	No Result	NO
175	A9WH42	No Result	No Result	transmem	Zinc binding	No Result	No Result	NO
176	A9WH43	No Result	No Result	coiled	EC3.6	No Result	No Result	NO
177	A9WH47	No Result	No Result	transmem	EC2.7	No Result	No Result	NO
178	A9WH50	No Result	No Result	signal	All lipid binding	No Result	No Result	NO
179	A9WH53	No Result	No Result	transmem	Iron binding	No Result	No Result	NO
180	A9WHN5	No Result	No Result	signal	TC2.A	No Result	No Result	NO
181	A9WHP3	No Result	No Result	transmem	TC3.A Primary	No Result	No Result	NO
182	A9WHQ3	CcmE superfamily	CcmE	transmem	All lipid binding	No Result	No Result	NO
183	A9WHQ4	CcmD_alt_fam	No Result	transmem	All lipid binding	Putativeccmoperon	No Result	NO
184	A9WHS0	No Result	No Result	No	Lipid binding	No Result	No Result	NO
185	A9WHS1	No Result	No Result	No	EC2.4	No Result	No Result	NO
186	A9WHS6	No Result	transmembrane	No	EC2.1	No Result	No Result	NO
187	A9WHT1	No Result	DUF444	No	Manganese	Uncharacterisedpro	No Result	NO
188	A9WHT4	No Result	No Result	signal	Copper binding	No Result	No Result	NO
189	A9WHU1	No Result	ABC2_membrane	transmem	EC2.5	Protein of	No Result	NO
190	A9WHU7	No Result	No Result	coiled	EC3.6	No Result	No Result	NO
191	A9WHV2	No Result	No Result	transmem	Zinc binding	No Result	No Result	NO
192	A9WHW0	No Result	MacB_PCD,FtsX	transmem	Zinc binding	MacB	No Result	NO
193	A9WHW5	No Result	No Result	No	All lipid binding	No Result	No Result	NO
194	A9WHW8	No Result	No Result	No	TC1.A	No Result	No Result	NO

195	A9WHW9	No Result	DUF2089,HTH_3	No	Sodium binding	Proteinofunknownf	No Result	NO
196	A9WHX0	No Result	No Result	No	Zinc binding	No Result	No Result	NO
197	A9WIE7	No Result	No Result	transmem	Chlorophyll	No Result	No Result	NO
198	A9WIF0	No Result	No Result	transmem	EC3.2	No Result	Glycosyl hydrolases	NO
199	A9WIF1	No Result	No Result	transmem	EC2.4	No Result	No Result	NO
200	A9WIF2	No Result	NTP_transf_4	No	Iron binding	UDP	No Result	NO
201	A9WIG5	No Result	No Result	transmem	Sodium binding	No Result	No Result	NO
202	A9WIG8	No Result	No Result	transmem	All lipid binding	No Result	No Result	NO
203	A9WIG9	No Result	No Result	No	Zinc binding	No Result	No Result	NO
204	A9WIH0	No Result	No Result	No	EC2.4	No Result	No Result	NO
205	A9WIH7	No Result	No Result	No	Zinc binding	No Result	No Result	NO
206	A9WII2	No Result	No Result	transmem	EC3.4	No Result	No Result	NO
207	A9WIJ4	No Result	No Result	No	All lipid binding	No Result	No Result	NO
208	A9WIJ5	No Result	SIMPL	signal	All lipid binding	Proteinofunknownf	No Result	NO
209	A9WIJ9	No Result	Polyketide_cyc2	transmem	Iron binding	Polyketidecyclase/	No Result	NO
210	A9WIK3	No Result	No Result	transmem	EC4.6 Lyases	No Result	No Result	NO
211	A9WIM7	No Result	No Result	transmem	Zinc binding	No Result	No Result	NO
212	A9WIM9	No Result	No Result	transmem	Zinc binding	No Result	No Result	NO
213	A9WJ54	No Result	No Result	transmem	TC3.A.3 Ptype	No Result	No Result	NO
214	A9WJ59	No Result	DUF4127	No	EC2.7	Proteinofunknownf	No Result	NO
215	A9WJ82	No Result	No Result	No	EC4.1 Lyases	No Result	No Result	NO
216	A9WJ83	No Result	GIDE	transmem	All lipid binding	E3 Ubiquitin	No Result	NO
217	A9WJ89	No Result	No Result	coiled	EC2.7	No Result	No Result	NO
218	A9WJ94	No Result	No Result	No	Metal binding	Carboxypeptidase	No Result	NO
219	A9WJ96	No Result	No Result	transmem	EC2.1	No Result	No Result	NO
220	A9WJ99	No Result	No Result	transmem	EC1.6	No Result	No Result	NO
221	A9WJB8	No Result	2Fe 2S_thioredx	No	Iron binding	Thioredoxin like	No Result	NO
222	A9WJC2	No Result	MacB_PCD,FtsX	No	Manganese	MacB like	No Result	NO
223	A9WJC7	No Result	No Result	signal	Zinc binding	No Result	No Result	NO
224	A9WJC8	No Result	No Result	No	All lipid binding	No Result	No Result	NO
225	A9WJE4	No Result	DUF4013	No	7	Protein of	No Result	NO
226	A9WJE5	No Result	DUF4013	No	EC3.6	Protein of	No Result	NO
227	A9WJE7	No Result	YtxH	No	All lipid binding	Uncharacterised	No Result	NO
228	A9WJX5	No Result	No Result	transmem	All lipid binding	No Result	No Result	NO
229	A9WJX9	No Result	No Result	transmem	All lipid binding	No Result	No Result	NO
230	A9WJY4	No Result	No Result	transmem	All lipid binding	No Result	No Result	NO
231	A9WK03	No Result	No Result	No	Metal binding	No Result	No Result	NO
232	A9WK07	No Result	No Result	No	All lipid binding	No Result	No Result	NO
233	A9WK09	No Result	No Result	signal	Iron binding	No Result	CD98 heavy chain,	NO
234	A9WK10	No Result	No Result	transmem	TC2.A	No Result	No Result	NO
235	A9WK15	No Result	No Result	coiled	All DNA	No Result	No Result	NO
236	A9WK16	No Result	No Result	transmem	All lipid binding	No Result	No Result	NO
237	A9WK25	No Result	No Result	No	Iron binding	No Result	No Result	NO
238	A9WK31	No Result	DUF503	No	EC2.7	Protein of	Hypothetical conserved	NO
239	A9WK33	No Result	DUF3782,	No	No Result	Protein of	No Result	NO
240	A9WK34	No Result	No Result	coiled	DNA directed	No Result	No Result	NO
241	A9WK41	No Result	Creatininase	No	TC3.A.3 P-type	Creatininase/forma	Creatinine	NO
242	A9WK45	No Result	No Result	transmem	EC2.4	No Result	No Result	NO
243	A9WK47	No Result	SBP_bac_11	transmem	EC1.1	Thiosulphate/Sulfat	No Result	NO
244	A9WK51	No Result	No Result	No	EC2.5	No Result	No Result	NO
245	A9WK68	No Result	No Result	transmem	TC3.A.5 Type II	No Result	No Result	NO
246	A9W9P1	No Result	DUF2064	No	Metal binding	Transferase 1,	2 phospho L lactate	NO
247	A9W9Q3	No Result	No Result	coiled	Zinc binding	No Result	No Result	NO
248	A9W9Q4	DUF1997	DUF1997	No	EC2.4	Protein of	No Result	NO
249	A9W9T0	Chlor_Arch_YYY	DUF2298	transmem	TC2.A	Protein of	No Result	NO
250	A9W9T2	TRX_Fd_family	No Result	No	EC4.2 Lyases	Thioredoxin like	Hydrogenase subunit	NO
251	A9W9T4	No Result	No Result	No	Iron binding	No Result	No Result	NO
252	A9W9V0	4Oxalocrotonate_	No Result	No	RNA binding	No Result	No Result	NO
253	A9W9V2	No Result	No Result	No	Zinc binding	No Result	No Result	NO
254	A9W9V9	No Result	No Result	signal	All lipid binding	No Result	No Result	NO
255	A9W9W3	CarboxypepD_reg	No Result	signal	All lipid binding	No Result	No Result	NO
256	A9W9W4	No Result	No Result	signal	Zinc binding	No Result	N acetylmuramoyl L	NO
257	A9W9X1	No Result	No Result	No	EC2.1	No Result	No Result	NO
258	A9W9X2	Ploop_NTPase	No Result	No	Zinc binding	No Result	No Result	NO
259	A9WA72	No Result	No Result	No	rRNA binding	No Result	No Result	NO
260	A9WA73	No Result	No Result	transmem	EC1.6	No Result	No Result	NO

261	A9WA75	No Result	No Result	No	Zinc binding	No Result	No Result	NO
262	A9WA79	No Result	No Result	signal	EC2.4	Six hairpin	No Result	NO
263	A9WA84	No Result	DUF429	No	Iron binding	Protein of	No Result	NO
264	A9WA96	No Result	No Result	transmem	7	No Result	No Result	NO
265	A9WAA0	No Result	No Result	transmem	G protein	No Result	No Result	NO
266	A9WAA3	No Result	No Result	transmem	7	No Result	No Result	NO
267	A9WAA6	Methyltransf_25	Methyltransf_31,	No	Zinc binding	No Result	UbiE/COQ5	NO
268	A9WAA7	PKS_KS	No Result	signal	EC3.2	No Result	No Result	NO
269	A9WAB4	Bac_rhamnosid	No Result	No	EC2.4	Six hairpin	No Result	NO
270	A9WAB6	No Result	No Result	signal	Iron binding	No Result	No Result	NO
271	A9WAB9	Csx15_IU	No Result	No	Structural	No Result	No Result	NO
272	A9WAC1	No Result	No Result	transmem	Zinc binding	No Result	No Result	NO
273	A9WAC2	No Result	YGGT	No	TC3.A.15 The	CCB3/YggT	No Result	NO
274	A9WAC3	No Result	No Result	signal	EC2.1	No Result	No Result	NO
275	A9WAC4	No Result	No Result	No	EC4.2 Lyases	No Result	No Result	NO
276	A9WAD2	No Result	No Result	No	EC2.1	No Result	No Result	NO
277	A9WAD4	No Result	No Result	transmem	TC2.A	No Result	No Result	NO
278	A9WAE6	No Result	No Result	signal	Zinc binding	No Result	No Result	NO
279	A9WAF0	No Result	No Result	transmem	No Result	No Result	No Result	NO
280	A9WAF1	Wzy_C	Wzy_C	transmem	Iron binding	O antigen ligase	No Result	YES
281	A9WAF2	No Result	No Result	signal	Zinc binding	No Result	No Result	NO
282	A9WAF4	No Result	No Result	No	No Result	No Result	No Result	NO
283	A9WAU1	NT_Polbeta-like	No Result	No	RNA binding	No Result	No Result	NO
284	A9WAU5	No Result	No Result	transmem	7	No Result	No Result	NO
285	A9WAV7	CofD_YvcK	UPF0052	transmem	EC1.9	LPPG:FO 2	Maternal effect embryo	YES
286	A9WAW0	No Result	No Result	No	Manganese	No Result	No Result	NO
287	A9WAW2	No Result	No Result	No	Metal binding	No Result	No Result	NO
288	A9WAX5	No Result	No Result	No	7	No Result	No Result	NO
289	A9WAX9	No Result	No Result	transmem	EC3.4	No Result	No Result	NO
290	A9WAY0	cl28280	EamA		TC2.A	No Result	No Result	NO
291	A9WAY2	No Result	No Result	No	Nickel binding	No Result	No Result	NO
292	A9WAY3	No Result	No Result	No	EC2.4	Alpha/Beta	No Result	NO
293	A9WAY4	No Result	No Result	signal	EC1.1	No Result	No Result	NO
294	A9WAY5	No Result	WXG100	No	TC3.A.15 The	Type VII secretion	No Result	NO
295	A9WB11	No Result	No Result	transmem	TC2.A	No Result	No Result	NO
296	A9WB12	DUF86	DUF86	No	TC4.A Group	Protein of	DNA polymerase, beta	NO
297	A9WB24	No Result	No Result	transmem	Iron binding	No Result	No Result	NO
298	A9WB25	No Result	No Result	transmem	EC3.6	No Result	No Result	NO
299	A9WB27	SpoIIM,ABC2_m	ABC2_membrane	No	TC2.A	ABC 2 family	No Result	NO
300	A9WBK2	PNPOx/FlaRed_li	F420H2_quin_red	No	All lipid binding	F420H(2)	no Result	NO
301	A9WBK6	DUF2892	DUF2892	No	7	Protein of	No Result	NO
302	A9WBK8	No Result	No Result	transmem	TC3.A.15 The	No Result	No Result	NO
303	A9WBL5	FlgD_ig	No Result	transmem	Iron binding	No Result	No Result	NO
304	A9WBM4	GluZincin	No Result	transmem	EC1.1	No Result	No Result	NO
305	A9WBM6	No Result	No Result	transmem	Zinc binding	No Result	No Result	NO
306	A9WBN0	SMC_N	No Result	transmem	Calcium binding	No Result	No Result	NO
307	A9WBQ0	Glyco_hydro_30	Glyco_hydro_cc	signal	All lipid binding	Glycoside	No Result	YES
308	A9WBQ5	No Result	No Result	signal	EC3.2	Glycoside	No Result	NO
309	A9WBQ6	No Result	No Result	signal	EC3.1	Glycoside	Putative 1,3 beta	NO
310	A9WBR1	DUF3592	DUF3592	No	EC1.9	Protein of	No Result	NO
311	A9WBR6	TIGR03847	DUF3090	No	Zinc binding	Protein of	No Result	NO
312	A9WBS6	No Result	No Result	No	EC2.4	No Result	No Result	NO
313	A9WBU0	Glyco_hydro_1	No Result	signal	EC2.4	Glycoside	CD98 heavy chain,	YES
314	A9WCC2	MMP_ACEL2062	Zincin_1	No	Iron binding	Zincin like	No Result	NO
315	A9WCC5	YtxH superfamily	No Result	transmem	EC4.1 Lyases	No Result	No Result	NO
316	A9WCC7	No Result	No Result	transmem	No Result	No Result	No Result	NO
317	A9WCD7	Peptidase_C11	Peptidase_C11	signal	Metal binding	Peptidase C11,	No Result	YES
318	A9WCE1	NT_PolNo	NTP_transf_5	No	All lipid binding	Uncharacterised	No Result	NO
319	A9WCE7	Trm11	No Result	No	EC2.4	S adenosyl L	Modification methylase	NO
320	A9WCE8	No Result	No Result	transmem	No Result	No Result	No Result	NO
321	A9WCE9	No Result	No Result	No	Magnesium	No Result	No Result	NO
322	A9WCF4	DUF512	DUF512	PDZ	EC1.1	PDZ superfamily	Stage IV sporulation	YES
323	A9WCF7	No Result	No Result	No	EC2.7	No Result	No Result	NO
324	A9WCG1	No Result	No Result	No	All lipid binding	No Result	No Result	NO
325	A9WCG8	No Result	No Result	coiled	EC2.7	No Result	No Result	NO
326	A9WCH2	No Result	No Result	coiled	Iron binding	No Result	No Result	NO

327	A9WCI1	No Result	No Result	No	All lipid binding	No Result	No Result	NO
328	A9WCI3	No Result	No Result	No	All lipid binding	No Result	No Result	NO
329	A9WCI6	No Result	No Result	No	Iron binding	P loop containing	No Result	NO
330	A9WD34	Sbt_1	Sbt_1	No	Zinc binding	Na+ dependent	No Result	NO
331	A9WD37	DUF3267	DUF3267	transmem	TC4.A Group	Putative zincin	No Result	YES
332	A9WD41	DUF3352	DUF3352	No	EC2.7	Protein of	No Result	NO
333	A9WD43	YecE	DUF72	No	Iron binding	Protein of	Putative uncharacterized	NO
334	A9WD56	No Result	No Result	transmem	EC3.6	No Result	No Result	NO
335	A9WD59	GlpC,Nitrate_red_	Nitrate_red_gam,	transmem	Iron binding	NarG like	NADPH dependent	YES
336	A9WD62	DUF4411	DUF4411	No	All lipid binding	Uncharacterised	No Result	NO
337	A9WD64	No Result	No Result	No	EC2.5	No Result	No Result	NO
338	A9WD65	DUF3800	DUF3800	No	All lipid binding	Protein of	No Result	NO
339	A9WD74	No Result	No Result	signal		No Result	No Result	NO
340	A9WD75	No Result	No Result	No	TC3.A.15 The	No Result	No Result	NO
341	A9WD78	No Result	No Result	No	EC3.8 Acting on	No Result	No Result	NO
342	A9WD79	No Result	No Result	transmem	TC9.B	No Result	No Result	NO
343	A9WD81	Radical_SAM	No Result	No	EC1.1	No Result	No Result	NO
344	A9WD96	DUF2256	DUF2256	No	Magnesium	Uncharacterised	No Result	NO
345	A9WD97	No Result	No Result	No	All DNA	No Result	No Result	NO
346	A9WDA0	No Result	No Result	transmem	TC1.C	No Result	No Result	NO
347	A9WDA9	No Result	Ferritin_2	transmem	Zinc binding	Twin arginine	No Result	NO
348	A9WDB1	RskA	RskA	No	Nuclear	Anti sigma K	No Result	NO
349	A9WDB6	No Result	No Result	No	RNA binding	No Result	No Result	NO
350	A9WDB7	No Result	No Result	transmem	EC6.2 Ligases	No Result	No Result	NO
351	A9WDB8	DNA_photolyase	No Result	No	copper binding	Cryptochrome/phot	No Result	NO
352	A9WDU0	No Result	No Result	transmem	Iron binding	No Result	No Result	NO
353	A9WDU9	WXG100,PH-like	WXG100	No	EC3.1	Type VII secretion	No Result	NO
354	A9WDV2	No Result	Glucosaminidase	transmem	Zinc binding	No Result	No Result	NO
355	A9WDV4	NTase_sub_bind	NTase_sub_bind	No	EC2.7	Nucleotidyltransfer	No Result	NO
356	A9WDW3	Exonuc_VII_L	coiled coil	No	All lipid binding	No Result	No Result	NO
357	A9WDW4	No Result	No Result	No	Zinc binding	No Result	No Result	NO
358	A9WDW6	No Result	No Result	transmem	Zinc binding	No Result	No Result	NO
359	A9WDW8	No Result	No Result	transmem	EC1.3	No Result	No Result	NO
360	A9WDW9	No Result	No Result	transmem	TC2.A	No Result	No Result	NO
361	A9WDX0	No Result	No Result	No	Zinc binding	No Result	No Result	NO
362	A9WDX1	No Result	No Result	transmem	EC1.6	No Result	No Result	NO
363	A9WDX2	No Result	No Result	signal	Copper binding	No Result	No Result	NO
364	A9WDX6	Stt3	No Result	transmem	copper binding	No Result	No Result	NO
365	A9WDX7	T2SSE_N	No Result	No	EC2.7	No Result	No Result	NO
366	A9WDZ5	No Result	No Result	coiled	Actin binding	No Result	No Result	NO
367	A9WE07	RhaT	EamA	No	Zinc binding	No Result	No Result	NO
368	A9WE09	BcsB superfamily	BcsB	signal	Metal binding	Cellulose synthase	No Result	YES
369	A9WE14	Heme_Cu_Oxidas	COX1	No	G protein	Cytochrome c	No Result	NO
370	A9WE15	No Result	No Result	transmem	TC3.A.15 The	No Result	No Result	NO
371	A9WEE4	TsaE	TsaE	No	Zinc binding	tRNA	ATPase, YjeE family	YES
372	A9WEE5	No Result	No Result	No	EC2.4	No Result	No Result	NO
373	A9WEF1	No Result	transmembrane	No	EC2.1	No Result	No Result	NO
374	A9WEG4	COG2308	CP_ATPgrasp_2,	No	EC2.4	Uncharacterised	No Result	NO
375	A9WEH8	DUF1524,DUF26	DUF262,DUF152	No	EC2.7	No Result	No Result	NO
376	A9WEI1	No Result	No Result	transmem	Manganese	No Result	No Result	NO
377	A9WEK0	No Result	No Result	signal	Calcium binding	No Result	No Result	NO
378	A9WEK1	PP2Cc	No Result	transmem	All lipid binding	No Result	No Result	NO
379	A9WEL0	Ras_like_GTPase	Arf	No	All lipid binding	Small GTPase	ADP ribosylation factor	YES
380	A9WEL3	ribokinase_pfkB_l	No Result	No	rRNA binding	No Result	No Result	NO
381	A9WEL7	No Result	No Result	No	Iron binding	No Result	No Result	NO
382	A9WEX4	SBBP,FN3	SBBP	FN3	EC3.2	Immunoglobulin	Genome polyprotein 2	YES
383	A9WEX6	Glyco_tranf_GTA	DUF2064	No	EC2.4	Transferase 1,	No Result	NO
384	A9WEZ5	DUF86	DUF86	No	EC4.2 Lyases	Protein of	Glr1165 protein	NO
385	A9WEZ7	DUF86	No Result	No	All lipid binding	No Result	No Result	NO
386	A9WEZ9	No Result	No Result	No	No Result	No Result	No Result	NO
387	A9WF20	Wzy_C	No Result	transmem	TC9.B	No Result	No Result	NO
388	A9WF32	CCG superfamily	Fer4_10,CCG,Fer	No	Iron binding	Glycolate oxidase,	Coenzyme F420	YES
389	A9WF36	No Result	No Result	transmem	Zinc binding	No Result	No Result	NO
390	A9WF48	DUF2085	DUF2085	transmem	Zinc binding	Protein of	No Result	NO
391	A9WF49	No Result	No Result	No	EC2.5	No Result	No Result	NO
392	A9WF55	No Result	No Result	No	EC1.1	No Result	No Result	NO

393	A9WF56	Atrophin1	No Result	No	Zinc binding	No Result	No Result	NO
394	A9WF57	No Result	No Result	No	TC3.A.3 P type	Protein of	No Result	NO
395	A9WF59	No Result	No Result	No	TC2.A.6	No Result	No Result	NO
396	A9WF63	No Result	No Result	No	All lipid binding	No Result	No Result	NO
397	A9WF64	No Result	No Result	No	All lipid binding	No Result	No Result	NO
398	A9WFN0	VgrG superfamily	No Result	No	All lipid binding	Vgr protein, OB	Rhs element Vgr protein	NO
399	A9WFN1	DUF4280	No Result	signal	Lipid binding	No Result	No Result	NO
400	A9WFN4	Baseplate_J	No Result	No	Zinc binding	Conserved	No Result	NO
401	A9WFN5	No Result	No Result	No	Zinc binding	No Result	No Result	NO
402	A9WFN6	DUF4815	No Result	No	All lipid binding	No Result	No Result	NO
403	A9WFN7	COG1233	No Result	coiled	EC2.7	No Result	No Result	NO
404	A9WFN9	Flot superfamily	No Result	transmem	TC2.A	No Result	No Result	NO
405	A9WFP2	No Result	No Result	No	EC2.1	No Result	No Result	NO
406	A9WFQ0	No Result	No Result	No	All lipid binding	No Result	No Result	NO
407	A9WFQ1	No Result	No Result	No	EC2.1	No Result	No Result	NO
408	A9WFQ2	mod_pep_cyc	No Result	No	EC1.2	Tetratricopeptide	No Result	NO
409	A9WFQ3	No Result	No Result	transmem	TC4.A Group	No Result	No Result	NO
410	A9WFR2	SDR_a8	Epimerase,NAD_	No	Iron binding	Epimerase family	3 beta hydroxysteroid	YES
411	A9WFT2	No Result	No Result	transmem	TC2.A	No Result	No Result	NO
412	A9WFT3	HSP70	No Result	coiled	All DNA	No Result	No Result	NO
413	A9WFU1	No Result	No Result	No	Zinc binding	No Result	No Result	NO
414	A9WFU4	PII superfamily	CdAMP_rec	No	All lipid binding	Cyclic di AMP	No Result	NO
415	A9WFU8	Mur_ligase_M	Mur_ligase_M,D	No	EC1.1	No Result	UDPN	NO
416	A9WFU9	Amidase	No Result	No	EC2.4	No Result	No Result	NO
417	A9WFV1	DUF5122,NHL	DUF5122	No	copper binding	Delta 60 repeat	No Result	NO
418	A9WFV5	No Result	No Result	No	EC2.7	No Result	No Result	NO
419	A9WFW1	No Result	No Result	No	EC2.1	No Result	No Result	NO
420	A9WFW3	No Result	No Result	No	TC3.A.15 The	No Result	No Result	NO
421	A9WFW6	No Result	CdAMP_rec	No	All lipid binding	Cyclic di AMP	No Result	NO
422	A9WGE6	Csx3_III	Cas_csx3	No	EC2.7	CRISPR associated	No Result	NO
423	A9WGE8	DUF1887	Cas_Cas02710	No	All lipid binding	Restriction	No Result	NO
424	A9WGE9	Cas10 superfamily	No Result	No	Zinc binding	No Result	No Result	NO
425	A9WGF2	RAMP_I_III	RAMPs	No	EC3.6	CRISPR type III	No Result	NO
426	A9WGF3	TIGR03984	No Result	No	Zinc binding	CRISPR associated	No Result	NO
427	A9WGF6	DUF3006	DUF3006	No	Sodium binding	Protein of	No Result	NO
428	A9WGG2	rhaM	rhaM	No	Iron binding	Rhamnose/fucose	L rhamnose mutarotase	YES
429	A9WGG9	RAMPs	RAMPs	No	Metal binding	CRISPR type III	No Result	NO
430	A9WGH1	No Result	No Result	No	Zinc binding	No Result	No Result	NO
431	A9WGH3	RAMP_I_III	RAMPs	No	All lipid binding	CRISPR type III	No Result	NO
432	A9WDJ0	UPF0158	UPF0158	No	EC3.1	Uncharacterised	No Result	NO
433	A9WDI8	No Result	No Result	transmem	TC9.B	No Result	No Result	NO
434	A9WDI7	Bactofilin,DUF34	No Result	transmem	TC4.A Group	No Result	No Result	NO
435	A9WDI2	No Result	No Result	transmem	TC2.A	No Result	No Result	NO
436	A9WDI0	RecG super	No Result	No	All lipid binding	No Result	No Result	NO
437	A9WDH7	DUF3623	DUF3623	transmem	TC2.A	Photosynthetic	No Result	YES
438	A9WDH1	GlyGly_RbtA	No Result	transmem	All lipid binding	No Result	Metabotropic glutamate	NO
439	A9WDG5	AAA_33	AAA_33	No	Iron binding	P loop containing	Adenylate kinase	YES
440	A9WDG4	No Result	No Result	transmem	EC3.2	Kelch type beta	No Result	NO
441	A9WDF3	DUF1641	DUF1641	coiled	Zinc binding	Protein of	No Result	NO
442	A9WDE9	No Result	No Result	No	rRNA binding	No Result	No Result	NO
443	A9WDE1	HTH_42	HTH_42	No	EC2.4	Protein of	No Result	NO
444	A9WDD2	No Result	No result	No	Metal binding	No Result	No Result	NO
445	A9WCS7	No Result	No Result	No	EC3.2	No Result	No Result	NO
446	A9WCR8	DUF4956	DUF4956	transmem	Zinc binding	Protein of	No Result	NO
447	A9WCR6	DUF507	DUF507	No	EC2.1	Protein of	No Result	NO
448	A9WCR1	No Result	No Result	No	EC2.5	No Result	No Result	NO
449	A9WCQ4	DUF2255	DUF2255	No	All lipid binding	Uncharacterised	No Result	NO
450	A9WCP3	No Result	No Result	No	Metal binding	No Result	No Result	NO
451	A9WCP0	TlyC	DUF21,CBS,Cor	transmem	TC9.B	FAD binding, type	Inner membrane protein	YES
452	A9WCN7	No Result	VCBS	transmem	All lipid binding	No Result	No Result	NO
453	A9WCN5	DUF4058	DUF4058	No	Iron binding	Protein of	No Result	NO
454	A9WCM9	No Result	No Result	no result	EC3.1	No Result	No Result	NO
455	A9WCM6	No Result	No Result	transmem	Zinc binding	No Result	No Result	NO
456	A9WCL6	5_nucleotid_C	No Result	transmem	TC3.A Primary	No Result	No Result	NO
457	A9WCL1	PPK2 super	PPK2	No	EC2.7	P loop containing	Probable thymidylate	YES
458	A9WCL0	No Result	No Result	transmem	TC2.A	Protein of	No Result	NO

459	A9WC09	No Result	No Result	transmem	Iron binding	No Result	No Result	NO
460	A9WC06	No Result	No Result	transmem	Zinc binding	No Result	No Result	NO
461	A9WC01	CE4_u7	No Result	No	EC2.4	No Result	No Result	NO
462	A9WBZ2	PMT_2 super	No Result	transmem	All lipid binding	No Result	No Result	NO
463	A9WBY1	AdoMet_MTases	No Result	transmem	TC2.A	S adenosyl L	Putative spermidine	YES
464	A9WBY0	No Result	No Result	transmem	EC2.4	Class I glutamine	No Result	NO
465	A9WBX5	No Result	No Result	transmem	EC3.2	No Result	No Result	NO
466	A9WBW6	DUF2892	DUF2892	transmem	Manganese	Protein of	No Result	NO
467	A9WBW2	No Result	No Result	transmem	TC2.A	No Result	No Result	NO
468	A9WBW0	metallo-	No Result	transmem	EC2.4	No Result	No Result	NO
469	A9WBV7	No Result	No Result	No	Zinc binding	No Result	No Result	NO
470	A9WBV4	GGN super family	No Result	transmem	TC2.A	No Result	No Result	NO
471	A9WBV1	No Result	No Result	transmem	TC2.A	No Result	No Result	NO
472	A9WBU6	Stt3 super	No Result	transmem	Zinc binding	No Result	No Result	NO
473	A9WBU5	No Result	No Result	transmem	TC4.A Group	No Result	No Result	NO
474	A9WBA3	DUF2442	DUF2442	No	All lipid binding	Protein of	LPXTG motif cell wall	NO
475	A9WBA2	DUF4160	DUF4160	No	TC3.A.5 Type II	Protein of	No Result	NO
476	A9WBA0	No Result	No Result	No	EC4.3 Lyases	Probable antitoxin	No Result	NO
477	A9WB99	No Result	No Result	No	All lipid binding	No Result	No Result	NO
478	A9WB96	No Result	No Result	No	Copper binding	No Result	No Result	NO
479	A9WB95	No Result	No Result	coiled	All lipid binding	No Result	No Result	NO
480	A9WB94	DUF2442	DUF2442	No	Sodium binding	Protein of	No Result	NO
481	A9WB93	DUF4160	DUF4160	No	Copper binding	Protein of	No Result	NO
482	A9WB92	No Result	No Result	No	Manganese	No Result	No Result	NO
483	A9WB49	No Result	No Result	No	Zinc binding	No Result	No Result	NO
484	A9WB85	No Result	No Result	No	EC2.1	No Result	No Result	NO
485	A9WB81	No Result	No Result	signal	Zinc binding	No Result	No Result	NO
486	A9WB83	No Result	No Result	No	Iron binding	No Result	No Result	NO
487	A9WB58	No Result	No Result	No	TC2.A.1 Major	No Result	Bifunctional protein	NO
488	A9WB89	GIYc	No Result	No	All lipid binding	No Result	No Result	NO
489	A9WB63	No Result	No Result	No	EC1.3	No Result	No Result	NO
490	A9WB84	RhsA super family	No Result	coiled	Zinc binding	No Result	No Result	NO
491	A9WB91	No Result	No Result	No	All lipid binding	No Result	No Result	NO
492	A9WB78	No Result	No Result	No	Iron binding	No Result	No Result	NO
493	A9WB87	No Result	No Result	No	EC2.4	No Result	No Result	NO
494	A9WB90	Imm26	Imm26	Pfam:Im	TC1.C	Immunity protein	No Result	YES
495	A9WB55	RhsA super family	No Result	No	TC1.C	No Result	No Result	NO
496	A9WB88	No Result	No Result	No	EC3.2	No Result	No Result	NO
497	A9WB76	No Result	No Result	transmem	EC2.1	No Result	No Result	NO
498	A9WB52	RhsA super family	No Result	coiled	Zinc binding	No Result	No Result	NO
499	A9WB48	No Result	No Result	No	Copper binding	No Result	No Result	NO
500	A9WB68	No Result	No Result	No	All DNA	No Result	No Result	NO
501	A9WB77	No Result	No Result	No	rRNA binding	No Result	No Result	NO
502	A9WB80	No Result	No Result	No	Iron binding	No Result	No Result	NO
503	A9WB82	No Result	No Result	No	Zinc binding	No Result	No Result	NO
504	A9WB79	No Result	No Result	signal	Zinc binding	No Result	No Result	NO
505	A9WB53	No Result	No Result	No	EC2.1	No Result	No Result	NO
506	A9WB51	No Result	No Result	No	Manganese	No Result	No Result	NO
507	A9WB75	RhsA super	No Result	No	EC2.4	No Result	No Result	NO
508	A9WAL5	No Result	DUF4058	No	EC2.7	Protein of	No Result	NO
509	A9WA40	No Result	PDDEXK_7	No	Zinc binding	PD (D/E)XK	No Result	NO
510	A9WAJ4	No Result	No Result	No	EC1.6	No Result	No Result	NO
511	A9WA20	No Result	No Result	transmem	Zinc binding	No Result	No Result	NO
512	A9WB33	No Result	DUF4230	transmem	EC2.7	Protein of	No Result	NO
513	A9WAK4	No Result	No Result	signal	All lipid binding	No Result	No Result	NO
514	A9WA08	No Result	Asp_protease_2	No	EC4.1 Lyases	Aspartic peptidase	No Result	NO
515	A9WAL4	No Result	DUF4058	No	EC1.18	Protein of	No Result	NO
516	A9WAH2	No Result	No Result	No	Iron binding	No Result	No Result	NO
517	A9WAJ9	No Result	No Result	No	EC2.7	No Result	No Result	NO
518	A9WKN5	DUF4177	No Result	No	EC4.1 Lyases	No Result	No Result	NO
519	A9WB47	PNo	No Result	No	EC2.1	No Result	No Result	NO
520	A9WKM2	DoxX superfamily	DoxX	transmem	Iron binding	DoxX family	No Result	YES
521	A9WAL2	No Result	No Result	No	All DNA	No Result	No Result	NO
522	A9WAH1	No Result	No Result	No	TC4.A Group	No Result	No Result	NO
523	A9WB37	No Result	LPG_synthase_T	transmem	Zinc binding	Lysylphosphatidylg	No Result	NO
524	A9WKL4	tRNA synt_2	No Result	transmem	Iron binding	No Result	No Result	NO

525	A9WA25	No Result	No Result	transmem	Zinc binding	No Result	No Result	NO
526	A9WAK0	No Result	No Result	transmem	All lipid binding	No Result	No Result	NO
527	A9WA60	No Result	No Result	transmem	Zinc binding	No Result	No Result	NO
528	A9WA39	No Result	No Result	signal	All lipid binding	No Result	No Result	NO
529	A9WA19	No Result	No Result	transmem	TC3.A.3 P type	No Result	No Result	NO
530	A9WAM0	No Result	No Result	signal	All lipid binding	No Result	No Result	NO
531	A9WAI6	No Result	No Result	No	EC4.1 Lyases	No Result	No Result	NO
532	A9WA55	No Result	No Result	No	All lipid binding	No Result	No Result	NO
533	A9WAJ5	No Result	No Result	No	All lipid binding	No Result	No Result	NO
534	A9WAI8	No Result	LPG_synthase_T	transmem	TC2.A	Lysylphosphatidylg	No Result	NO
535	A9WA43	No Result	Reprolysin_4	signal	All lipid binding	Anti sigma K	No Result	NO
536	A9WAL7	No Result	No Result	No	Zinc binding	No Result	No Result	NO
537	A9WA56	No Result	DUF488	No	Zinc binding	Protein of	No Result	NO
538	A9WKL2	Ploop_NTPase	No Result	No	All lipid binding	No Result	LuxR family	NO
539	A9WAK2	No Result	LPG_synthase_T	transmem	TC2.A	Lysylphosphatidylg	No Result	NO
540	A9WAK6	No Result	ABC2_membrane	transmem	TC9.B	ABC 2 family	No Result	NO
541	A9WA51	No Result	EI24	transmem	EC1.10	No Result	No Result	NO
542	A9WKL0	DUF2237	DUF2237	No	EC2.4	Protein of	No Result	NO
543	A9WAG9	No Result	No Result	transmem	Iron binding	No Result	No Result	NO
544	A9WA42	No Result	Glyco_hydro_42,	transmem	EC3.2	Flagellar filament	CD98 heavy chain,	YES
545	A9WJ36	No Result	DUF1501	signal	EC3.1	No Result	No Result	NO
546	A9WJT8	No Result	No Result	transmem	G protein	Domain of	No Result	NO
547	A9WIY8	No Result	No Result	No	All lipid binding	No Result	No Result	NO
548	A9WJ48	No Result	No Result	No	EC2.5	No Result	No Result	NO
549	A9WJ41	No Result	ABC2_membrane	transmem	Iron binding	Protein of	No Result	NO
550	A9WJW7	No Result	No Result	No	RNA binding	No Result	No Result	NO
551	A9WJW9	No Result	No Result	No	Iron binding	No Result	No Result	NO
552	A9WJR0	No Result	No Result	transmem	Sodium binding	No Result	No Result	NO
553	A9WJV7	No Result	PD40	transmem	All lipid binding	No Result	Tricorn protease	NO
554	A9WJ52	No Result	No Result	transmem	TC9.B	No Result	No Result	NO
555	A9WJ42	No Result	ABC2_membrane	transmem	TC9.A	Protein of	No Result	NO
556	A9WJ01	No Result	DUF445	transmem	EC3.6	Protein of	No Result	NO
557	A9WJQ3	No Result	Frankia_peptide	No	TC3.A.5 Type II	Ribosomally	Nitrile hydratase alpha	NO
558	A9WKH2	No Result	No Result	transmem	TC3.A.15 The	No Result	No Result	NO
559	A9WKH1	No Result	No Result	No	Calcium binding	No Result	No Result	NO
560	A9WID3	No Result	No Result	No	Zinc binding	No Result	No Result	NO
561	A9WJT6	No Result	No Result	transmem	EC1.9	No Result	No Result	NO
562	A9WJT9	No Result	SpoIIAA like	No	Iron binding	No Result	No Result	NO
563	A9WJS8	No Result	No Result	No	Iron binding	No Result	No Result	NO
564	A9WKJ8	No Result	No Result	transmem	Manganese	No Result	No Result	NO
565	A9WIC2	No Result	DUF2085	transmem	Iron binding	Protein of	No Result	NO
566	A9WIB7	No Result	No Result	No	EC3.1	No Result	No Result	NO
567	A9WIZ8	No Result	No Result	transmem	All DNA	XdhC CoxI	No Result	NO
568	A9WIB6	No Result	No Result	No	Magnesium	No Result	No Result	NO
569	A9WJT5	No Result	DUF1444	No	EC2.7	Protein of	No Result	NO
570	A9WJW8	No Result	No Result	No	EC4.1 Lyases	No Result	No Result	NO
571	A9WJV1	No Result	Cas_Cas02710	transmem	EC4.1 Lyases	No Result	No Result	NO
572	A9WIE0	No Result	Bactofilin	No	TC2.A	Bactofilin A/B	No Result	NO
573	A9WIY3	No Result	LPG_synthase_T	transmem	TC2.A	Lysylphosphatidylg	No Result	NO
574	A9WJR6	No Result	No Result	transmem	EC4.1 Lyases	No Result	No Result	NO
575	A9WIB2	No Result	No Result	No	Iron binding	No Result	No Result	NO
576	A9WI81	No Result	No Result	transmem	EC3.6	Carboxypeptidase	No Result	NO
577	A9WJP7	No Result	No Result	No	EC2.7	No Result	No Result	NO
578	A9WJ37	No Result	DUF1800	transmem	EC1.1	Protein of	No Result	NO
579	A9WIA5	No Result	No result	transmem	All lipid binding	Protein of	No Result	NO
580	A9WKJ9	No Result	No Result	transmem	TC2.A	No Result	No Result	NO
581	A9WJT0	No Result	No Result	No	EC4.2 Lyases	No Result	No Result	NO
582	A9WKK7	No Result	RE_Bpu10I	No	EC1.3	Restriction	No Result	NO
583	A9WJ25	No Result	No Result	No	All lipid binding	No Result	No Result	NO
584	A9WIC6	No Result	No Result	No	EC2.5	HDIG domain	No Result	NO
585	A9WIZ3	No Result	No Result	coiled	EC2.4	No Result	No Result	NO
586	A9WJP6	No Result	No Result	signal	EC6.4 Ligases	No Result	No Result	NO
587	A9WKJ6	No Result	No Result	transmem	EC1.7	No Result	No Result	NO
588	A9WI91	No Result	No Result	transmem	Zincbinding	No Result	No Result	NO
589	A9WIB4	No Result	No Result	No	Zinc binding	No Result	No Result	NO
590	A9WJT3	No Result	YscW	signal	EC2.1	YscW/ExsB	No Result	NO

591	A9WID9	No Result	No Result	No	Zinc binding	No Result	No Result	NO
592	A9WI84	No Result	No Result	No	Iron binding	No Result	No Result	NO
593	A9WIB0	No Result	No Result	No	Magnesium	No Result	No Result	NO
594	A9WHF5	No Result	No Result	No	No Result	No Result	No Result	NO
595	A9WHK9	No Result	DUF1772	transmem	EC2.4	No Result	No Result	NO
596	A9WHH6	No Result	No Result	No	No Result	No Result	No Result	NO
597	A9WHF4	No Result	No Result	transmem	TC3.A.5 Type II	No Result	No Result	NO
598	A9WHK4	No Result	No Result	transmem	All lipid binding	No Result	No Result	NO
599	A9WHH0	No Result	No Result	No	All lipid binding	ClpP/crotonase like	No Result	NO
600	A9WIY1	No Result	No Result	transmem	TC2.A	Phosphatidic acid	No Result	NO
601	A9WHL7	No Result	No Result	transmem	7	No Result	No Result	NO
602	A9WHK5	No Result	No Result	No	Metal binding	Protein of	No Result	NO
603	A9WI71	No Result	No Result	No	All DNA	No Result	No Result	NO
604	A9WHK6	No Result	No Result	transmem	Iron binding	No Result	No Result	NO
605	A9WHM6	No Result	No Result	transmem	EC2.4	WD40/YVTN	Sensor histidine	NO
606	A9WHM0	No Result	No Result	signal	EC3.2	Putative auto	No Result	NO
607	A9WHK2	No Result	No Result	No	EC3.1	Integrase/recombin	No Result	NO
608	A9WHL0	No Result	DUF2269	transmem	All lipid binding	No Result	No Result	NO
609	A9WHH5	No Result	FeS_assembly_P	No	EC2.4	Putative zinc or	No Result	NO
610	A9WHL6	No Result	DUF2568	transmem	TC9.A	Protein of	No Result	NO
611	A9WGU7	DUF951	DUF951	No	Sodium binding	Protein of	No Result	NO
612	A9WHG1	No Result	DUF3445	No	EC2.4	Protein of	No Result	NO
613	A9WGV0	RhaT superfamily	EamA	transmem	TC2.A	EamA domain	No Result	YES
614	A9WGT2	No Result	No Result	No	All lipid binding	EamA domain	No Result	NO
615	A9WHI6	No Result	No Result	transmem	EC3.1	No Result	No Result	NO
616	A9WGR8	No Result	No Result	No	All lipid binding	No Result	No Result	NO
617	A9WHF3	DUF5115	No Result	FN3,sign	All lipid binding	Immunoglobulin	No Result	NO
618	A9WHI4	No Result	No Result	transmem	EC1.1	Immunoglobulin	No Result	NO
619	A9WHL5	No Result	No Result	No	No Result	No Result	No Result	NO
620	A9WHI8	No Result	No Result	transmem	TC2.A	No Result	No Result	NO
621	A9WJM1	No Result	DUF4058	No	EC1.14	No Result	No Result	NO
622	A9WJN9	No Result	No Result	No	TC3.A.5 Type II	No Result	No Result	NO
623	A9WHZ7	DUF4491	DUF4491	transmem	EC2.1	No Result	No Result	NO
624	A9WG40	No Result	No Result	transmem	All lipid binding	Protein of	No Result	NO
625	A9WI60	No Result	DUF3095	No	EC1.1	Protein of	No Result	NO
626	A9WG43	No Result	SDH_sah,CLP_pr	signal	EC2.7	Peptidase S49,	Signal peptide peptidase	YES
627	A9WIV2	No Result	No Result	No	Zinc binding	No Result	No Result	NO
628	A9WKB4	TctC	TctC	signal	EC1.1	No Result	Probable extra	NO
629	A9WFY0	No Result	No Result	signal	All lipid binding	Bordetella uptake	Fis family transcriptional	NO
630	A9WIT5	No Result	No Result	transmem	TC2.A.1 Major	No Result	No Result	NO
631	A9WK99	DUF1641	DUF1641	No	EC2.7	Protein of	No Result	NO
632	A9WKA1	Lamp superfamily	No Result	signal	All lipid binding	No Result	No Result	NO
633	A9WG45	No Result	No Result	No	Zinc binding	Glycoside	No Result	NO
634	A9WF79	No Result	No Result	No	RNA binding	No Result	No Result	NO
635	A9WHY4	COG1470	NPCBM_assoc	signal	Zinc binding	Alpha	Receptor type tyrosine	YES
636	A9WIT2	No Result	No Result	transmem	All lipid binding	Glyoxalase/Bleomy	No Result	NO
637	A9WJI0	No Result	No Result	No	mRNA binding	No Result	No Result	NO
638	A9WKB3	TctB	TctB	transmem	TC2.A	Protein of	No Result	NO
639	A9WF71	No Result	No Result	No	EC2.7	No Result	No Result	NO
640	A9WG12	No Result	DUF3054	transmem	TC2.A	Protein of	No Result	NO
641	A9WJG3	No Result	No Result	No	EC3.1	No Result	No Result	NO
642	A9WGN5	No Result	No Result	transmem	Zinc binding	No Result	No Result	NO
643	A9WJK2	No Result	AAA_21,15,DUF	No	Zinc binding	No Result	ABC transporter, ATP	NO
644	A9WFX3	No Result	FGE sulfatase	No	Zinc binding	No Result	No Result	NO
645	A9WG41	No Result	Polyketide_cyc2	No	Nuclear	Polyketide	No Result	NO
646	A9WJN6	No Result	No Result	transmem	TC1.C	No Result	No Result	NO
647	A9WGP3	No Result	APA3_viroporin	transmem	TC3.A Primary	No Result	No Result	NO
648	A9WIN8	No Result	TPR_14	transmem	EC2.7	No Result	No Result	NO
649	A9WKA3	DUF1461	No Result	signal	Manganese	No Result	No Result	NO
650	A9WFD3	DUF1998	DUF1998	No	Metal binding	DEAD/DEAH box	No Result	NO
651	A9WFY5	No Result	LPAM_1	signal	All lipid binding	Uncharacterised	No Result	NO
652	A9WEM3	No Result	LPAM_1	signal	EC2.4	No Result	No Result	NO
653	A9WF75	SBBP	SBBP	FN3,tran	EC2.7	No Result	ABC transporter,	NO
654	A9WJL2	No Result	ABC2_membrane	transmem	EC2.7	No Result	No Result	NO
655	A9WI50	No Result	No Result	transmem	RNA binding	No Result	No Result	NO
656	A9WIT7	Beta lactamase2	Beta lactamase2	signal	All lipid binding	No Result	Extended spectrum beta	NO

657	A9WFC8	No Result	No Result	signal	All lipid binding	Six bladed beta	Protein TolB	NO
658	A9WIR8	No Result	No Result	signal	All lipid binding	No Result	No Result	NO
659	A9WKF0	No Result	No Result	No	Copper binding	No Result	No Result	NO
660	A9WKF4	No Result	RskA	transmem	Iron binding	No Result	No Result	NO
661	A9WIW6	PD40	No Result	signal	All lipid binding	Six bladed beta	Tricorn protease homolog	YES
662	A9WJL6	No Result	Neurensin	transmem	EC2.1	Six bladed beta	No Result	NO
663	A9WK91	No Result	Baculo_LEF 10	No	EC2.7	No Result	No Result	NO
664	A9WJM0	No Result	No Result	signal	All lipid binding	No Result	No Result	NO
665	A9WFX5	No Result	No Result	No	EC2.4	No Result	No Result	NO
666	A9WJH9	No Result	No Result	No	Actin binding	No Result	No Result	NO
667	A9WKB2	TctA superfamily	TctA	transmem	All lipid binding	No Result	No Result	NO
668	A9WFA7	No Result	No Result	signal	EC2.4	Protein of	No Result	NO
669	A9WJJ0	No Result	No Result	No	Iron binding	No Result	No Result	NO
670	A9WI63	No Result	TrkA_C	No	EC2.7	No Result	No Result	NO
671	A9WGP1	DUF3352	DUF3352	transmem	Zinc binding	Protein of	No Result	NO
672	A9WKB6	K_oxygenase	K_oxygenase	No	Iron binding	No Result	tRNA 5	NO
673	A9WK77	No Result	No Result	No	EC3.1	No Result	No Result	NO
674	A9WI49	No Result	DUF3792	transmem	EC3.6	Protein of	No Result	NO
675	A9WFX6	No Result	No Result	No	Iron binding	No Result	No Result	NO
676	A9WG31	PhnG	PhnG	No	EC3.1	No Result	No Result	NO
677	A9WG24	No Result	No Result	transmem	EC1.6	P loop containing	No Result	NO
678	A9WJK7	No Result	Bac_export_3	transmem	All lipid binding	No Result	Sulfur relay protein TusC	NO
679	A9WKC9	HAS	DUF87	No	EC2.5	Helicase HerA like	No Result	NO
680	A9WG39	No Result	GHMP_kinases_	No	Zinc binding	Ribosomal protein	L arabinokinase	NO
681	A9WG20	No Result	zf Paramyx P	No	EC5.3	No Result	Tequila, isoform	NO
682	A9WIR2	BNR	Sortilin Vps10	No	Zinc binding	No Result	No Result	NO
683	A9WIT6	No Result	Med9	coiled	EC3.6	No Result	Precorrin 2 C20	NO
684	A9WJP1	Frankia_peptide	Frankia_peptide	No	Magnesium	Ribosomally	No Result	NO
685	A9WHZ1	DUF3329	PhoR	transmem	EC2.4	Phosphate regulon	No Result	YES
686	A9WFY9	DUF4352	DUF4352	signal	EC2.4	No Result	No Result	NO
687	A9WK85	No Result	PhdYeFM_antito	No	EC1.15	Immunoprotective	Antitoxin phd	NO
688	A9WGQ7	No Result	DUF3120	No	Zinc bindingc	No Result	No Result	NO
689	A9WF90	HATPase_c	No Result	No	EC2.5	No Result	Multi sensor hybrid	NO
690	A9WJM2	DUF4058	No Result	No	EC1.14	No Result	No Result	NO
691	A9WI44	Na_Pi_cotrans	Na_Pi_cotrans	transmem	Manganese	No Result	Putative uncharacterized	NO
692	A9WFD7	No Result	CTP_transf_like	No	EC2.7	Sodium dependent	Probable nicotinate	NO
693	A9WF74	No Result	No Result	No	All lipid binding	No Result	No Result	NO
694	A9WF69	DUF4938	DUF4938	No	Manganese	Protein of	No Result	NO
695	A9WIW5	No Result	No Result	No	Manganese	No Result	No Result	NO
696	A9WF91	CHAT,SIR2_2	CHAT	No	EC3.6	No Result	No Result	NO
697	A9WGN7	No Result	No Result	transmem	Manganese	No Result	No Result	NO
698	A9WJK3	No Result	Patatin	No	EC3.1	Restriction	No Result	NO
699	A9WIR1	No Result	No Result	No	All lipid binding	Beta grasp domain	No Result	NO
700	A9WIU6	No Result	Thioredoxin	transmem	TC1.C	No Result	Soluble secreted antigen	NO
701	A9WJP3	No Result	No Result	No	EC2.7	No Result	No Result	NO
702	A9WF77	LppX_LprAFG	LppX_LprAFG	signal	All lipid binding	No Result	No Result	NO
703	A9WJM5	DUF294,cNMP_b	DUF294	cNMP,C	EC3.6	RmlC like jelly roll	cAMP dependent protein	YES
704	A9WK83	No Result	No Result	transmem	EC1.6	No Result	No Result	NO
705	A9WI33	No Result	DUF3397	transmem	Zinc binding	No Result	No Result	NO
706	A9WKE5	No Result	No Result	signal	EC3.2	Fibronectin type III	No Result	NO
707	A9WG23	No Result	Transport_MerF	transmem	7	No Result	No Result	NO
708	A9WFA9	No Result	No Result	No	7	No Result	No Result	NO
709	A9WG44	No Result	No Result	No	All lipid binding	No Result	No Result	NO
710	A9WJH1	No Result	Beta_helix	transmem	All lipid binding	Pectin lyase	No Result	NO
711	A9WI39	No Result	DUF4440	transmem	All lipid binding	No Result	No Result	NO
712	A9WK82	Calx beta	Calx beta	transmem	Zinc binding	No Result	No Result	NO
713	A9WFC5	No Result	No Result	No	All lipid binding	No Result	No Result	NO
714	A9WJF9	AI 2E_transport	AI 2E_transport	transmem	TC2.A	Transmembrane	No Result	YES
715	A9WJI3	No Result	Glyco_hydro_16	No	EC2.4	No Result	No Result	NO
716	A9WI55	Trns_repr_metal	Trns_repr_metal	No	rRNA binding	Metal sensitive	No Result	NO
717	A9WJI4	DUF2085	No Result	transmem	Copper binding	Protein of	No Result	NO
718	A9WK76	No Result	No Result	No	Zinc binding	Peptidase MA like	No Result	NO
719	A9WF99	No Result	No Result	signal	TC3.A.3 P type	No Result	No Result	NO
720	A9WHX6	DUF1428	DUF1428	No	Zinc binding	Protein of	No Result	NO
721	A9WHX5	DUF4256	DUF4256	No	EC2.4	Protein of	No Result	NO
722	A9WHB3	No Result	DUF4342	No	EC2.4	No Result	No Result	NO

723	A9WGH8	Cas_NE0113	Cas_NE0113	No rseult	EC3.4	CRISPR assoc	No Result	NO
724	A9WHD8	No Result	No Result	No	Iron binding	No Result	No Result	NO
725	A9WHD5	No Result	tRNA_anti codon	signal	All lipid binding	No Result	No Result	NO
726	A9WHC9	Asp B	Uma2	coiled	TC3.D Primary	Restriction	Uncharacterized protein	YES
727	A9WGH9	DUF1887,	DUF1887	No	All lipid binding	Protein of	No Result	NO
728	A9WGN0	No Result	Cellulase	transmem	All lipid binding	No Result	No Result	NO
729	A9WHC1	No Result	No Result	transmem	EC1.9	No Result	No Result	NO
730	A9WGI1	No Result	DUF2070	transmem	Zinc binding	No Result	No Result	NO
731	A9WGI2	No Result	No Result	No	All lipid binding	No Result	No Result	NO
732	A9WHB2	No Result	MRF_C1	No	EC2.7	No Result	No Result	NO
733	A9WHA0	No Result	ATP synt_B	coiled	Sodium binding	No Result	No Result	NO
734	A9WHA1	DUF177	YceD	No	Iron binding	No Result	No Result	NO
735	A9WHX4	DUF2200	DUF2200	No	Zinc binding	Uncharacterised	Uncharacterized protein	NO
736	A9WHB5	No Result	No Result	transmem	Iron binding	No Result	No Result	NO
737	A9WH59	No Result	No Result	transmem	All lipid binding	No Result	No Result	NO
738	A9WGH6	No Result	No Result	No	rRNA binding	No Result	No Result	NO
739	A9WGH5	RAMPs	RAMPs	No rseult	Zinc binding	No Result	No Result	NO
740	A9WGM0	No Result	No Result	No	Zinc binding	No Result	No Result	NO
741	A9WGF0	RAMPs	No Result	No	EC2.4	No Result	No Result	NO
742	A9WHE7	Abhydrolase_6,Se	Abhydrolase_6	No	Zinc binding	Putative hydrolase	No Result	NO
743	A9WGL3	No Result	MBD	No	EC2.5	No Result	No Result	NO
744	A9WGI0	No Result	No Result	signal	Zinc binding	No Result	No Result	NO
745	A9WGI8	No Result	No Result	No	Magnesium	No Result	No Result	NO
746	A9WGM3	LTD	LTD	signal	Zinc binding	Lamin tail domain	Lamin C, isoform B	YES
747	A9WGL2	No Result	No Result	transmem	Iron binding	Lamin tail domain	No Result	NO
748	A9WGM1	No Result	zf CCCH_6	No	Calcium binding	No Result	No Result	NO
749	A9WGH4	TIGR03984	No Result	No	Iron binding	No Result	No Result	NO
750	A9WCB5	DUF333	DUF333	signal	All lipid binding	Protein of	No Result	NO
751	A9WAT0	COG1543 super	Glyco_hydro_57,	No	EC2.4	1,4 alpha glucan	1,4 alpha glucan	YES
752	A9WAS8	No Result	No Result	No	7	No Result	No Result	NO
753	A9WAS7	No Result	No Result	No	All lipid binding	No Result	No Result	NO
754	A9WAS5	GRDB super	GRDB	No	Zinc binding	Selenoprotein B,	No Result	NO
755	A9WAS2	CP_ATPgrasp_2		No	Iron binding	No Result	No Result	NO
756	A9WAR6	No Result	No Result	No	EC2.7	No Result	No Result	NO
757	A9WAR5	YIH1	UPF0029,DUF19	No	EC3.8 Acting on	No Result	No Result	NO
758	A9WAR1	No Result	No Result	No	EC4.6 Lyases	No Result	No Result	NO
759	A9WAQ7	No Result	No Result	transmem	All lipid binding	No Result	No Result	NO
760	A9WAP6	AdoMet_MTases	Methyltransf_19	No	Iron binding	No Result	No Result	NO
761	A9WAP5	lysS super family	No Result	No	EC1.9	No Result	No Result	NO
762	A9WAN6	PerM	AI 2E_transport	transmem	TC2.A	Transmembrane	No Result	YES
763	A9WA62	DUF2227	DUF2227	transmem	All lipid binding	Protein of	No Result	NO
764	A9WA21	No Result	No Result	No	All DNA	No Result	No Result	NO
765	A9W9N7	DUF92	DUF92	transmem	EC3.6	Protein of	No Result	NO
766	A9WBC8	No Result	No Result	transmem	Iron binding	No Result	No Result	NO
767	A9WBC5	No Result	No Result	coiled	7	No Result	No Result	NO
768	A9WBC3	No Result	RE_SacI	No	EC2.7	Restriction	No Result	NO
769	A9WBB6	No Result	No Result	No	Iron binding	No Result	No Result	NO
770	A9WBB5	No Result	No Result	No	EC2.4	No Result	No Result	NO
771	A9WBB3	No Result	No Result	No	Copper binding	No Result	No Result	NO
772	A9WBA9	YbbP super	MacB_PCD ,FtsX	transmem	TC2.A	No Result	No Result	NO
773	A9WBA5	No Result	No Result	No	All lipid binding	No Result	No Result	NO
774	A9WBI7	No Result	No Result	transmem	EC4.1 Lyases	No Result	No Result	NO
775	A9WCP7	Fe_III_red_FhuF	FhuF	No	All lipid binding	Ferric iron	No Result	NO
776	A9WDR7	No Result	No Result	No	All lipid binding	No Result	No Result	NO
777	A9WEV5	DUF3341	DUF3341	transmem	EC3.5	Protein of	No Result	NO
778	A9WHG8	2A78 super family	EamA	transmem	TC2.A	No Result	No Result	NO
779	A9WHZ6	DUF4491	DUF4491	transmem	Zinc binding	Protein of	No Result	NO
780	A9WIB1	No Result	No Result	No	Zinc binding	No Result	No Result	NO
781	A9WIH9	PRK09752 super	No Result	transmem	All lipid binding	No Result	No Result	NO
782	A9WJJ6	YGGT	YGGT	transmem	7	CCB3/YggT	No Result	YES
783	A9WJR9	No Result	No Result	No	Metal binding	No Result	No Result	NO
784	A9WK72	No Result	No Result	No	Iron binding	No Result	No Result	NO
785	A9WKG3	No Result	No Result	transmem	TC9.B	No Result	No Result	NO
