# Supplementary material for: Deciphering the functional role of hypothetical proteins from Chloroflexus aurantiacs J-10-f1 using bioinformatics approach
Source: Mol Biol Res Commun. 2020 Sep;9(3):129–39. doi: 10.22099/mbrc.2020.36894.1495 (PMC7727763; doi:10.22099/mbrc.2020.36894.1495)
Supplement: Supplementary Material 3 [file mbrc-9-129-s003.pdf]

Table S3: Sub-Cellular Localization and Transmembrane Characterization of HC-HPs

UniProt ID	Subcellular localization					Trans membrane helices prediction		
	Psortb	PSLpred	CELLO	SecretomeP	SignalP	HMMTOP	TMHMM	SOSUI
A9WC42	Cytoplasmic	Cytoplasmic	Cytoplasmic	No	No	0	0	Soluble
A9WC50	Cytoplasmic Membrane	Cytoplasmic	Inner Membrane	No	No	3	2	Soluble
A9WC89	Cytoplasmic Membrane	Inner-membrane	Cytoplasmic	No	No	0	0	Soluble
A9WCX1	Cytoplasmic	Cytoplasmic	Membrane	No	No	3	2	Membrane
A9WD08	Cytoplasmic Membrane	Inner-membrane	Membrane	No	No	7	7	Membrane
A9WDN5	Unknown	Extracellular	Extracellular	Yes	No	0	0	Soluble
A9WE66	Cytoplasmic Membrane	Inner-membrane	Inner Membrane	No	No	10	9	Membrane
A9WE95	Cytoplasmic Membrane	Inner-membrane	Inner Membrane	No	No	5	5	Membrane
A9WEQ6	Unknown	Extracellular	Extracellular	Yes	Yes	1	0	Soluble
A9WAF1	Cytoplasmic	Inner-membrane	Cytoplasmic	No	No	13	11	Membrane
A9WAV7	Cytoplasmic Membrane	Inner-membrane	Periplasmic /Cytoplasmic	No	No	5	2	Membrane
A9WBQ0	Cytoplasmic Membrane	Cytoplasmic	Outer Membrane/ Cytoplasmic	No	Yes	1	0	Membrane
A9WCD7	Unknown	Periplasmic	Inner Membrane/ Cytoplasmic	No	Yes	0	1	Membrane
A9WCF4	Cytoplasmic	Cytoplasmic	Outer Membrane/InnerMembrane	No	No	0	0	Soluble
A9WD37	Cytoplasmic Membrane	Inner-membrane	Cytoplasmic	No	No	4	4	membrane
A9WD59	Cytoplasmic Membrane	Cytoplasmic	Inner Membrane	No	No	6	6	Membrane
A9WE09	Cytoplasmic Membrane	Periplasmic	Inner Membrane/ Cytoplasmic	No	Yes	1	1	Membrane
A9WEE4	Cytoplasmic	Cytoplasmic	Cytoplasmic	No	No	0	0	Soluble
A9WEL0	Cytoplasmic	Cytoplasmic	Cytoplasmic	No	No	0	0	Soluble
A9WEX4	Extracellular	Extracellular	Inner Membrane	Yes	No	0	0	Soluble
A9WF32	Cytoplasmic Membrane	Cytoplasmic	Extracellular/ Outer Membrane	No	No	0	0	Soluble
A9WFR2	Cytoplasmic	Cytoplasmic	Inner Membrane	No	No	0	0	Soluble
A9WGG2	Unknown	Cytoplasmic	Cytoplasmic	No	No	0	0	Soluble
A9WDH7	Cytoplasmic Membrane	Inner-membrane	Inner Membrane	No	No	6	6	Membrane
A9WDG5	Cytoplasmic	Cytoplasmic	Cytoplasmic	No	No	0	0	Soluble
A9WCP0	Cytoplasmic Membrane	Cytoplasmic	Inner Membrane	No	Yes	3	4	Membrane
A9WCL1	Cytoplasmic	Cytoplasmic	Cytoplasmic	No	No	0	0	Soluble
A9WBY1	Cytoplasmic Membrane	Inner-membrane	Membrane	No	No	13	14	Membrane
A9WBA3	Unknown	Cytoplasmic	Cytoplasmic	No	No	0	0	Soluble
A9WB90	Cytoplasmic	cytoplasmic	Cytoplasmic	No	No	10	0	Soluble
A9WKM2	Cytoplasmic Membrane	inner membrane	Outer Membrane	No	No	0	3	Membrane
A9WA42	Unknown	Periplasmic	Outer Membrane	Yes	Yes	0	1	Membrane
A9WGV0	Cytoplasmic Membrane	Inner membrane	OuterMembrane	No	No	10	10	Membrane
A9WG43	Cytoplasmic Membrane	Cytoplasmic	Cytoplasmic	No	Yes	1	0	Membrane

A9WHY4	Unknown	periplasmic	Cytoplasmic	Yes	Yes	2	2	Membrane
A9WIW6	Cytoplasmic Membrane	Periplasmic	InnerMembrane	Yes	Yes	1	1	Membrane
A9WHZ1	Cytoplasmic Membrane	Inner-membrane	Membrane	No	No	1	1	Membrane
A9WJM5	Cytoplasmic	Cytoplasmic	Cytoplasmic	No	No	1	0	Soluble
A9WJF9	Cytoplasmic Membrane	Inner-membrane	Membrane	No	No	7	8	Membrane
A9WGM3	Cytoplasmic Membrane	Cytoplasmic	Periplasmic/Cytoplasmic	No	No	1	0	Soluble
A9WGM3	Cytoplasmic Membrane	cytoplasmic	Periplasmic/ Cytoplasmic	No	No	1	0	Soluble
A9WAT0	Cytoplasmic	Cytoplasmic	Cytoplasmic	No	No	0	0	Soluble
A9WAN6	Cytoplasmic Membrane	Inner-membrane	Membrane	No	No	7	8	Membrane
A9WJJ6	Cytoplasmic Membrane	Inner-membrane	Membrane	No	Yes	2	2	Membrane

For HMMTOP and TMHMM ‘0’ indicate the no membrane helices is predicted.
